# HIV-1 adaptation studies reveal a novel Env-mediated homeostasis mechanism for evading lethal hypermutation by APOBEC3G

**DOI:** 10.1371/journal.ppat.1007010

**Published:** 2018-04-20

**Authors:** Terumasa Ikeda, Menelaos Symeonides, John S. Albin, Ming Li, Markus Thali, Reuben S. Harris

**Affiliations:** 1 Department of Biochemistry, Molecular Biology, and Biophysics, University of Minnesota, Minneapolis, Minnesota, United States of America; 2 Institute for Molecular Virology, University of Minnesota, Minneapolis, Minnesota, United States of America; 3 Howard Hughes Medical Institute, University of Minnesota, Minneapolis, Minnesota, United States of America; 4 Cellular, Molecular and Biomedical Sciences Graduate Program and Department of Microbiology and Molecular Genetics, Larner College of Medicine and College of Agriculture and Life Sciences, University of Vermont, Burlington, Vermont, United States of America; Emory University, UNITED STATES

## Abstract

HIV-1 replication normally requires Vif-mediated neutralization of APOBEC3 antiviral enzymes. Viruses lacking Vif succumb to deamination-dependent and -independent restriction processes. Here, HIV-1 adaptation studies were leveraged to ask whether viruses with an irreparable *vif* deletion could develop resistance to restrictive levels of APOBEC3G. Several resistant viruses were recovered with multiple amino acid substitutions in Env, and these changes alone are sufficient to protect Vif-null viruses from APOBEC3G-dependent restriction in T cell lines. Env adaptations cause decreased fusogenicity, which results in higher levels of Gag-Pol packaging. Increased concentrations of packaged Pol in turn enable faster virus DNA replication and protection from APOBEC3G-mediated hypermutation of viral replication intermediates. Taken together, these studies reveal that a moderate decrease in one essential viral activity, namely Env-mediated fusogenicity, enables the virus to change other activities, here, Gag-Pol packaging during particle production, and thereby escape restriction by the antiviral factor APOBEC3G. We propose a new paradigm in which alterations in viral homeostasis, through compensatory small changes, constitute a general mechanism used by HIV-1 and other viral pathogens to escape innate antiviral responses and other inhibitions including antiviral drugs.

## Author summary

APOBEC3G is a virus restriction factor that blocks the replication of Vif-deficient HIV-1 by deamination-dependent and -independent mechanisms. The HIV-1 accessary protein Vif counteracts APOBEC3G through a proteasome-mediated degradation pathway. However, viruses often possess multiple distinct mechanisms to evade innate immune responses, and it was unknown whether HIV-1 possesses alternative mechanisms for escaping restriction by APOBEC3G. To investigate this possibility, HIV-1 with a non-revertable *vif* deletion was adapted in stepwise cultures to increasing amounts of APOBEC3G. Three independent APOBEC3G resistant viral isolates acquired amino acid substitutions in Env. Mechanistic studies showed that these Env adaptations cause decreased fusogenicity, which re-optimizes viral homeostasis by allowing increased Gag-Pol packaging and higher rates of reverse transcription, which in turn protect viral DNA from lethal hypermutation by APOBEC3G. Thus, these results demonstrate a novel Env-dependent mechanism mediated by RT that HIV-1 can utilize to escape APOBEC3G-mediated restriction. Sequence comparisons suggest that transmitting isolates may also utilize this mechanism. More broadly, our results suggest a new paradigm in which relatively small changes in essential viral processes and overall viral homeostasis can have rather large phenotypic consequences such as enabling resistance to potent antiviral measures.

## Introduction

The seven members of the human APOBEC3 (A3) protein family are DNA cytosine deaminases encoded by tandemly arranged genes on chromosome 22 [1, 2]. These enzymes restrict the replication of a broad number of retroviruses including HIV-1 and some DNA viruses, and inhibit the mobilization of several endogenous retroelements and retrotransposons (reviewed by [3–6]). Of seven A3 proteins, only four—A3D, A3F, A3G, and A3H (stable haplotypes only)—contribute to HIV-1 restriction in primary T cells ([7–10] and references therein). These A3s inhibit HIV-1 replication by packaging into the viral particles through an RNA-dependent mechanism and, upon entry into new target cells, physically interfering with the progression of reverse transcription and deaminating single-stranded viral cDNA cytosines to uracils (reviewed by [3–5]). Viral cDNA uracils often become immortalized through reverse transcription, as these nucleobases template the insertion of genomic strand adenines that ultimately become G-to-A mutations. Such mutational events can alter the function of viral components or inactivate the virus.

HIV-1 encodes an accessory protein, viral infectivity factor (Vif), to counteract the antiviral activity of cellular A3s. HIV-1 Vif recruits an E3 ligase complex comprised of CBF-β, CUL5, ELOB, ELOC, and RBX2 to target restrictive A3s for proteasome-mediated degradation (reviewed by [3–5]). Vif also downregulates the expression of these *A3* genes by binding the transcription co-factor CBF-β [11]. In cases where Vif is expressed sufficiently in virus-producing cells, HIV-1 will counteract restriction via the aforementioned mechanisms. However, if amounts of restrictive A3s exceed the capacity of Vif, these A3s can be packaged into viral particles and inhibit HIV-1 replication in the next target cell.

Although Vif-mediated proteasomal degradation is a widely accepted mechanism for counteracting restriction by cellular A3 proteins, a Vif-independent mechanism may exist as an alternative for lentiviruses such as HIV-1 or as a primary mechanism for retroviruses/elements that lack a Vif-like system. As such, we have hypothesized that HIV-1 has a Vif-independent secondary A3 evasion mechanism [12–14]. Here, we directly tested this hypothesis by adapting Vif-null HIV-1 variants to be able to robustly replicate in the presence of restrictive levels of A3G. Interestingly, three adapted viral isolates that emerged from these step-wise selections had each acquired multiple amino acid substitutions in the envelope glycoprotein (Env). These changes still allowed packaging of restrictive levels of A3G into the viral particles but they prevented A3G from causing destructive levels of hypermutation following entry into T cells. Molecular clones with adapted Env exhibit lower fusogenic activity, increased Gag-Pol packaging, and faster rates of reverse transcription following entry into target T cells, which causes proportional decreases in A3G deamination and G-to-A hypermutation. These studies combine to demonstrate a novel homeostatic mechanism in which alterations in essential Env and RT activities allow Vif-null HIV-1 to resist restriction by A3G.

## Results

### Vif-null HIV-1 can adapt to replicate in the presence of restrictive levels of A3G

**Fig 1:**
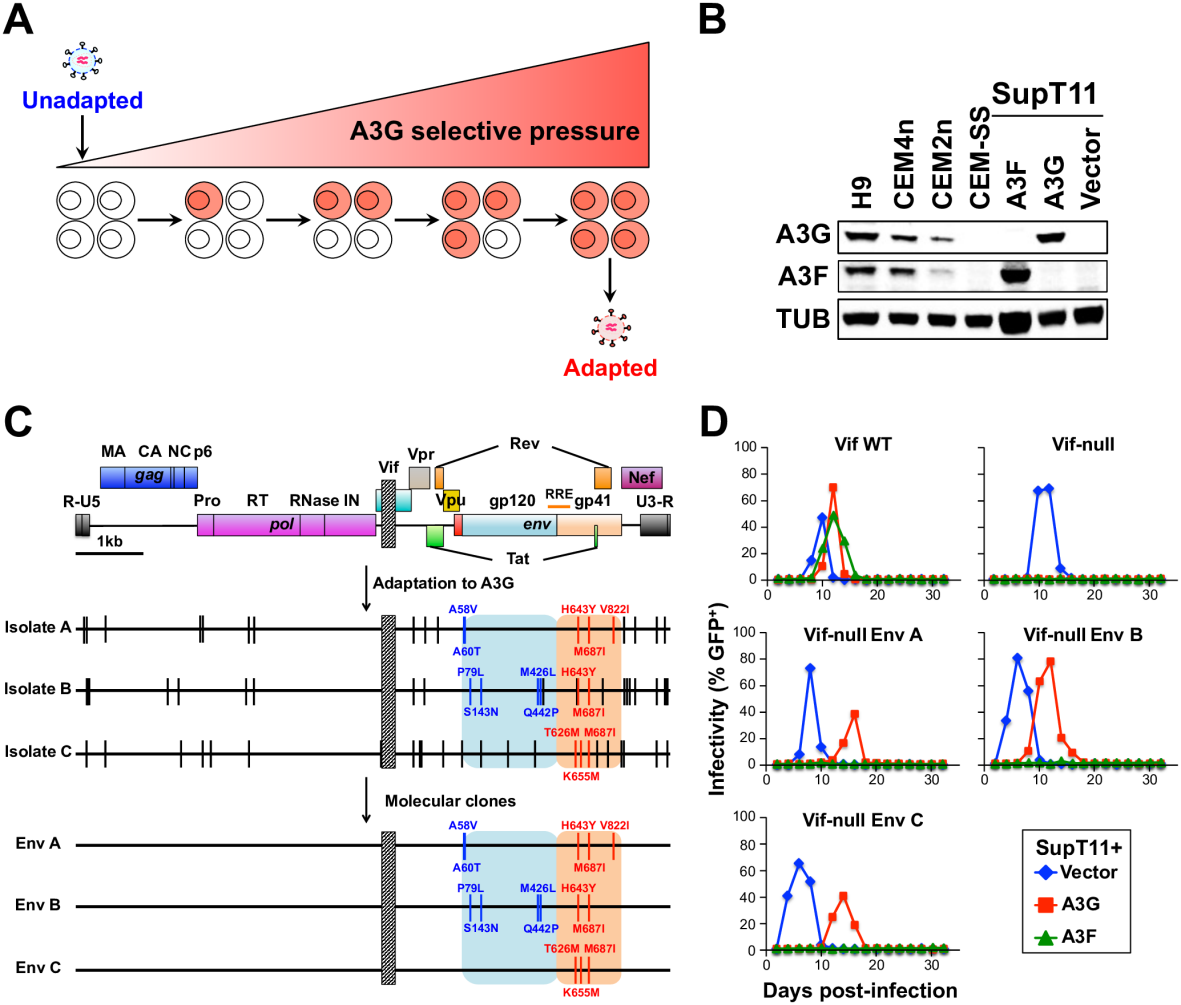
Env mutations render Vif-null HIV-1 fully resistant to A3G. (A) A schematic of the stepwise procedure used for virus adaptation to A3G. (B) Immunoblots of endogenous or stably expressed A3G or A3F in the indicated cell lines. Tubulin (TUB) is the loading control. (C) Schematic of the *vif*-null parental virus, the 3 independent A3G-adapted isolates, and the 3 molecular clones. Vertical bars depict fixed mutations, and a box depicts the *vif* deletion. The gp120 and gp41 regions of *env* are highlighted in blue and red. (D) Representative spreading infection data for the indicated viral molecular clones in SupT11 cells stably expressing a vector control, A3G, or A3F.

An HIV-1 strain with an irreparable 230 bp deletion in *vif* was subjected to stepwise passages with increased proportions of SupT11 T cells stably expressing restrictive levels of A3G (Fig 1A and 1B). Viruses that emerged from this initial selection procedure were subjected to an additional round of step-wise selection using CEM2n cells, which express restrictive levels of endogenous A3G and A3F ([8] and see immunoblot in Fig 1B). Three independent A3G resistant isolates emerged from these selection experiments, and each showed strong resistance to A3G as well as to the related enzyme A3F (S1A Fig). PCR analyses showed that the original 230 bp *vif* deletion remained intact (S1B Fig).

To identify mutations responsible for the Vif-independent resistance phenotype, we sequenced overlapping fragments spanning the full proviral DNA genome. This analysis revealed an accumulation of G-to-A mutations ranging from 3.2 to 3.4 per kb with the majority occurring in an A3G-preferred context, 5’-GG-to-AG, consistent with the antiviral enzyme itself promoting adaptation (S1C Fig). All A3G resistant isolates acquired mutations in various viral ORFs including *gag*, *pol*, *vpr*, *rev*, *env*, and *nef* (schematic in Fig 1C and full list of mutations in S1 Fig legend). The *env* gene had the greatest number of missense mutations (5 to 9 per isolate), and each isolate had at least 3 changes in the region encoding gp41 (orange-shaded region in Fig 1C). Isolates A and B share H643Y, and all three isolates share M687I, suggesting a common resistance mechanism. Several of these Env amino acid substitutions occur in the C-terminal heptad repeat region of gp41 (H643Y, T626M, and K655M), and most of the resistance-conferring substitutions match residues observed in transmitted/founder and chronic HIV-1 isolates [15]. Additionally, amino acid positions 58 and 79 in gp120, shown previously to associate structurally with gp41 [16], were also changed in the resistant isolates. This clustering of amino acid substitutions to Env suggested a common underlying molecular mechanism. Hereafter, these changes are grouped and referred to as Env A (A58V, A60T, H643Y, M687I, and V822I), Env B (P79L, S143N, M426L, Q442P, H643Y, and M687I), and Env C (T626M, K655M, and M687I) (Fig 1C).

### Env adaptations in Vif-null HIV-1 confer full resistance to A3G

To determine whether the selected sets of *env* mutations alone confer resistance to A3G, we constructed *vif*-null molecular clones with each combination of adaptive *env* mutations, produced viral stocks by transfection into 293T cells, and analyzed replication kinetics using SupT11 cells stably expressing A3G, A3F, or an empty control vector (representative data in Fig 1D and summary of independent experiments in S1 Table). All viruses, including Vif-proficient and Vif-null parental stocks, replicated with similar kinetics in SupT11 cells expressing the empty control vector. This result demonstrated that the adaptive changes in Env caused no overt replication defect. In addition, each Vif-null Env variant replicated robustly in A3G-expressing SupT11 cells with comparable but slightly delayed kinetics relative to the Vif-proficient parental virus. As expected, the replication of the Vif-null parental virus with no adaptive Env substitutions was fully restricted by A3G under the same conditions. Similar results were obtained using an independent T cell line, CEM-SS, stably expressing restrictive A3G levels, indicating that the resistance mechanism is not simply a peculiarity of the SupT11 T cell system (representative data in S2A and S2B Fig and summary of independent experiments in S2 Table).

Although the original adapted viral isolates showed resistance to both A3G and A3F, the Vif-null molecular clones carrying each set of Env amino acid substitutions were only resistant to A3G (Fig 1D and S1 Table). As above, this result was reproducible in CEM-SS cells stably expressing restrictive levels of A3G or A3F (S2A–S2D Fig and S2 Table). Therefore, a different combination of adaptive changes in the original selected viral isolates are likely to confer A3F resistance, most likely acquired in part during the second round of step-wise selection using CEM2n. These data indicate that the HIV-1 restriction mechanisms of A3G and A3F are genetically distinct, as inferred previously [12–14, 17]. However, most importantly, these data combine to demonstrate that the adaptive Env changes are alone sufficient to confer a strong Vif-like resistance to A3G-mediated restriction. Accordingly, the following studies are dedicated to elucidating this unanticipated Env-mediated molecular mechanism.

### Cell-cell fusion and virus transmission characteristics of Vif-null HIV-1 and derivatives with Env adaptations

**Fig 2:**
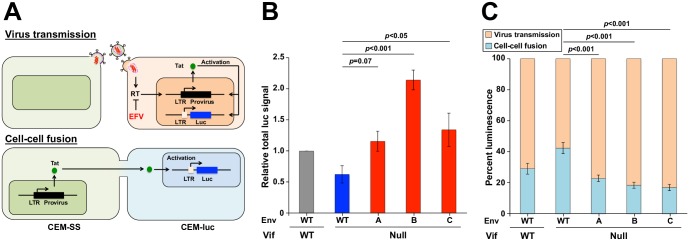
Env mutations exhibit reduced syncytium formation and foster more efficient virus transmission. (A) Schematic of virus transmission and cell-cell fusion events that result in luciferase expression upon co-culture of HIV-infected CEM-SS producer cells and CEM-luc target cells. In each scenario, HIV-1 Tat drives expression of an LTR-luciferase reporter gene in the CEM-luc target cells. Virus transmission is sensitive to RT inhibition by EFV because infection and *de novo* expression of Tat is required, whereas cell-cell fusion is resistant to EFV because preexisting Tat from an infected CEM-SS cell is able to activate reporter gene expression. (B) Total luminescence normalized to Gag expression and reported relative to the Vif-proficient virus. Each histogram bar represents the mean +/- SEM of the normalized data from 4 biologically independent experiments (*p*-values above each panel from one-way ANOVA and Fisher’s LSD test). (C) Luminescence signal attributable to cell-cell fusion or virus transmission for the indicated viruses. Cell-cell fusion events are quantified as the fraction of total luciferase signal that is resistant to EFV-treatment, and virus transmission events are quantified by subtracting the cell-cell fusion signal from the total luminescence signal. Each histogram bar represents the mean +/- SEM of 4 biologically independent experiments (*p*-values above each panel from one-way ANOVA and Fisher’s LSD test).

The amino acid substitutions common to all three Env variants occur within the C-terminal heptad repeat region of gp41, which is crucial for Env’s function as a fusogen (reviewed by [18]). To test the possibility that these adaptive changes modulate Env’s fusogenic activity in T cell cultures, a luciferase-based assay was used to quantify syncytium formation and virus transmission ([19]; schematic in Fig 2A). In this assay, luciferase activity is quantified in the presence and absence of the RT inhibitor efavirenz (EFV). EFV blocks luciferase activity from virus transmission events but not from cell-cell fusion events. Thus, the amount of syncytium formation (cell-cell fusion) corresponds directly to the luciferase signal upon EFV treatment, and this is genuine fusion because it is fully suppressed by the fusion inhibitor peptide C34 (S3 Fig). The amount of virus transmission is determined by subtracting the luciferase signal with EFV from the total luciferase signal obtained without EFV.

Interestingly, the total luciferase activity of the three adapted Env variants ranged from 2- to 4-fold higher than that of the Env wild-type (WT), Vif-null parental virus (i.e., the pre-adapted virus), and they were equivalent (Env A and Env C) or greater (Env B) than those of the original Vif-proficient virus (Fig 2B). These total luciferase activities were comprised of lower levels of cell-cell fusion and proportionately higher levels of transmission (Fig 2C). The relative distribution of these activities more closely resembled that of the original Vif-proficient virus, but it is important to note that the adapted Envs showed a significant 50% reduction in cell-cell fusion activity (p<0.001; blue bars in Fig 2C). Similar cell-cell fusion phenotypes were evident in a HeLa cell-based split GFP reconstitution assay (S4 Fig), and in experiments assaying the adapted Env variants within a Vif-proficient context (S5 Fig). At this time we do not have a molecular explanation for why Vif deficiency alone perturbs the fusogenic activities of Env, but the adaptive changes in Env clearly overcome this issue by decreasing the fusogenic activity of Env and increasing transmission events (i.e., restoration of homeostasis).

### Env adaptations protect Vif-null HIV-1 from A3G-catalyzed DNA deamination in T cells

We next asked if the A3G resistant phenotype could be recapitulated in single-cycle virus infection assays using 293T as producer cells in the presence of varying levels of exogenous A3G. The molecular clone of HIV-1 used for these experiments expresses a wild-type or an adapted Env and the 293T producer cells do not express the viral receptor CD4 and, therefore, syncytium formation in these cells will be rare. After virus production, cell-free virus-containing supernatants are recovered and used to infect CEM-GFP reporter cells, again minimizing opportunities for syncytium formation. Under these model conditions, the infectivity of the Vif-null Env variants is restricted by A3G nearly as well as the parental Vif-null virus (S6 Fig). Therefore, the benefit conferred by Env adaptations is lost in this model system, which supports the idea that decreased syncytium formation and/or increased transmission is part of the resistance mechanism in T cells.

**Fig 3:**
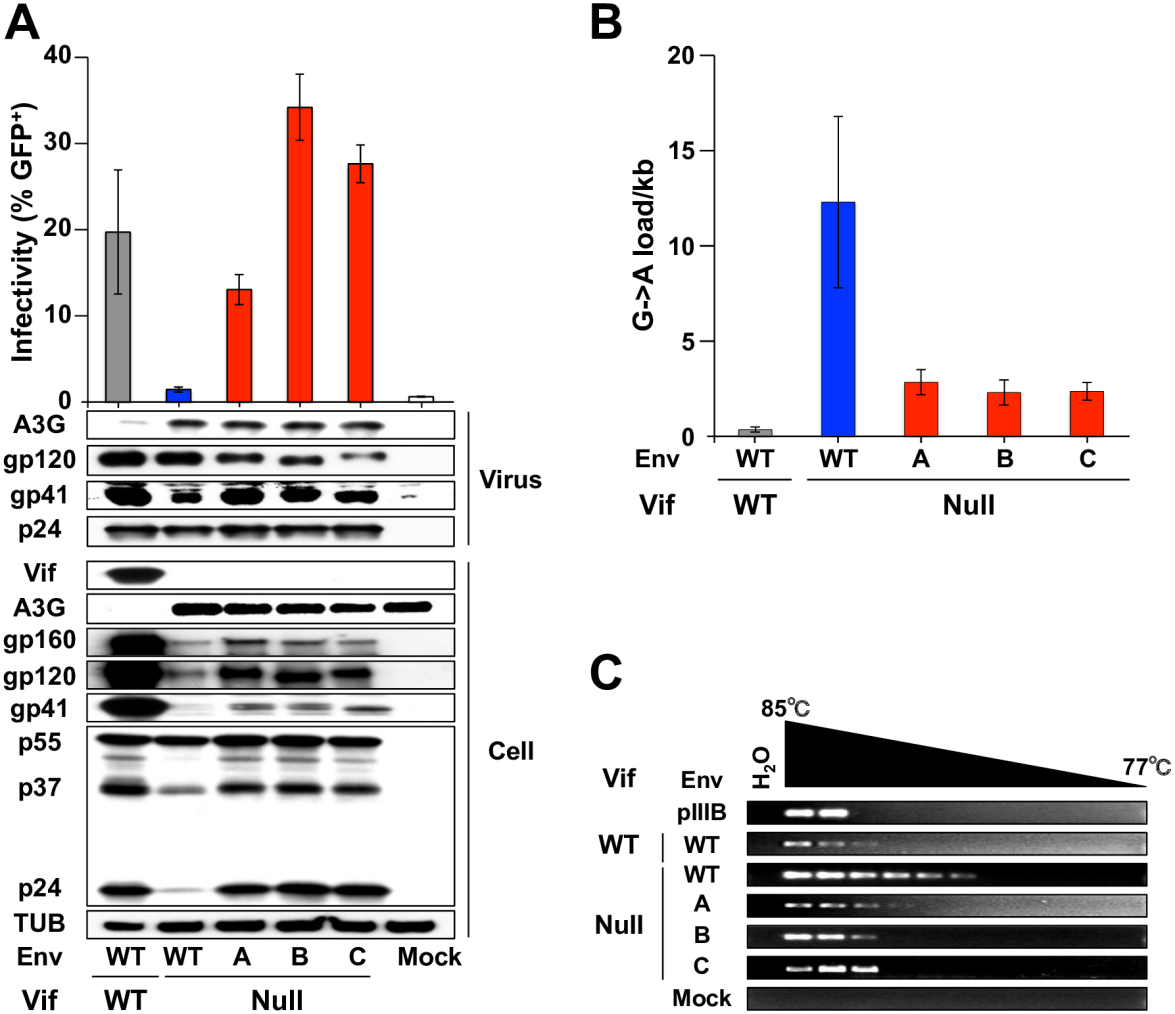
Env adaptations protect Vif-null HIV-1 from A3G deamination. (A) Representative pseudo-single cycle infectivity data for the indicated viruses produced in SupT11 cells stably expressing A3G. Infectivity data report the average +/- SD (n = 3). Immunoblots of the indicated proteins in viral particles following p24 normalization and producer cells are shown below for one representative experiment. (B) G-to-A mutation loads in proviral DNA from viruses originally produced in SupT11 cells expressing A3G (mean +/- SD of 3 independent experiments with a minimum of 10 sequences or 5,640 bp analyzed per condition). (C) Images of ethidium bromide-stained agarose gels containing *pol* 3D-PCR products recovered from CEM-GFP cells infected with the indicated viruses produced in SupT11-A3G cells. Untransfected proviral plasmid (pIIIB) and DNA from uninfected CEM-GFP (mock) are controls.

To directly test this idea in a T cell line which expresses the viral receptors, and thus where syncytium formation can take place during virus production (unlike in 293Ts), VSV-G pseudotyped HIV-1 was used to infect SupT11 cells expressing A3G or a vector control and nascent viruses produced from the infected T cell lines were harvested 48 h later, titered using the CEM-GFP system, and subjected to immunoblotting (Fig 3A). Consistent with spreading infection results, the Vif-null Env variants displayed infectivity levels comparable to Vif-proficient virus in SupT11 cells expressing A3G, yet packaged A3G levels were still indistinguishable from those in the Vif-null parental virus particles (viral particle immunoblots in Fig 3A). Additionally, despite restrictive levels of A3G packaging, A3G-mediated hypermutation was dramatically reduced in proviral DNAs of the Env variants as evidenced by data obtained from sequencing viral fragments from high-fidelity PCR amplifications and from 3D-PCR analyses of the *pol* region (Fig 3B and 3C and S7 Fig). Taken together with the results presented above, these data combine to show that the Env amino acid substitutions and consequent reduced syncytium formation somehow protect Vif-null HIV-1 from catastrophic levels of DNA deamination despite restrictive levels of A3G packaging.

### Intravirion A3G localization and activity are unaffected by Env adaptations

**Fig 4:**
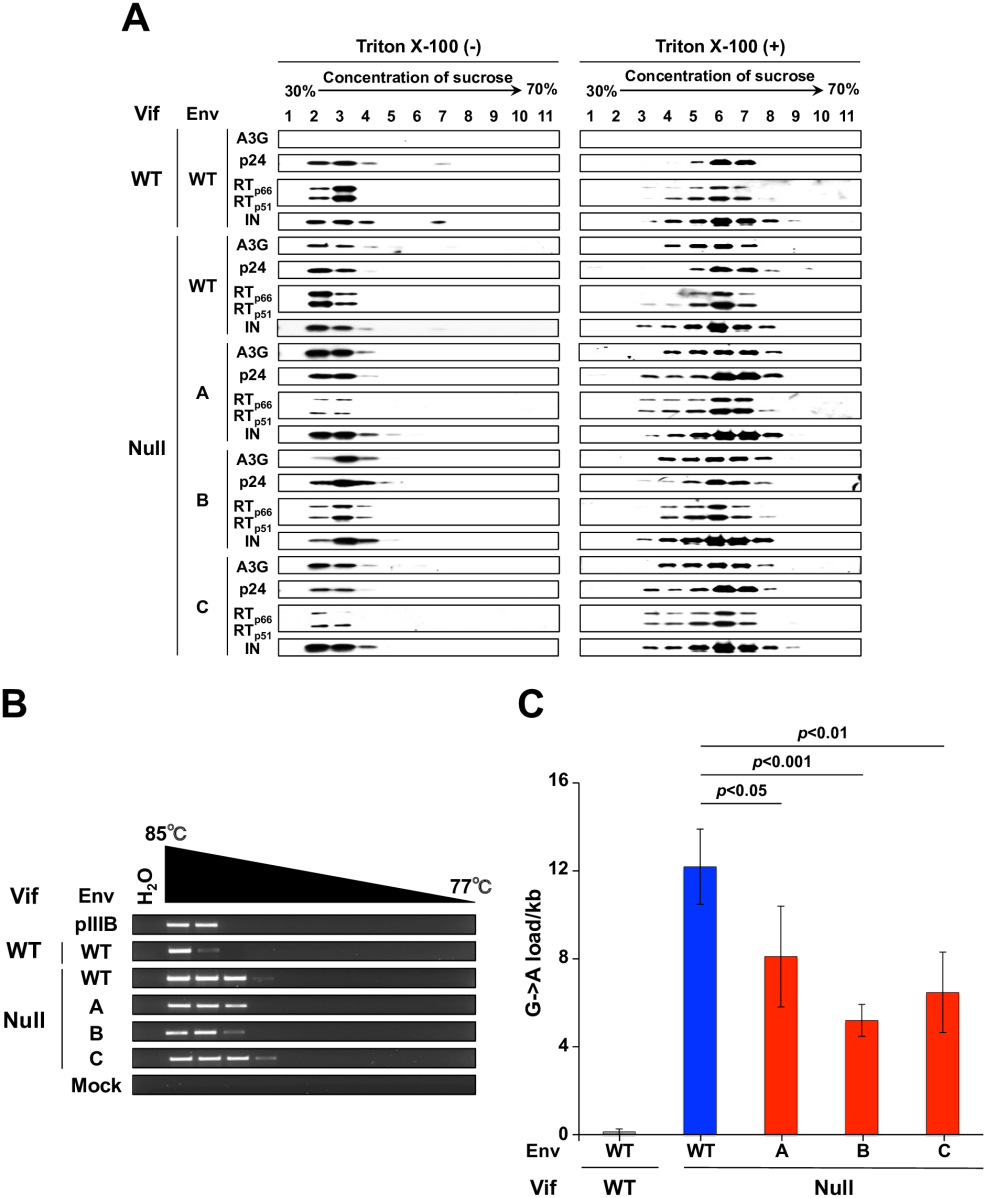
Env adaptations partly inhibit the capacity of packaged A3G to deaminate viral cDNA. (A) Immunoblots of viral particles of the indicated genotypes produced in SupT11-A3G cells and fractionated by ultracentrifugation through a sucrose gradient in the presence or absence of detergent. A3G co-sediments with viral core components under all conditions except the Vif-proficient scenario where it is efficiently degraded. (B) Representative images of *pol* 3D-PCR products using viral cDNAs subjected to ERT. The indicated viruses were originally produced in SupT11 cells expressing A3G. (C) G-to-A mutation loads of high-fidelity, high temperature *pol* gene amplicons from viruses originally produced in SupT11-A3G and subjected to ERT (mean +/- SD of independent 4 experiments with a minimum of 10 sequences or 5,640 bp analyzed per condition). Statistical comparisons were done using a one-way ANOVA and Fisher’s LSD test (*p*-values above each panel).

A3G is able to restrict Vif-deficient HIV-1 in part because it localizes to the core of nascent particles, and this is thought to occur through a Nucleocapsid (NC)-mediated interaction with packaged RNAs [20–26]. Since Env is targeted to assembling viral particles through an interaction between the cytoplasmic tail of the gp41 domain and the Matrix (MA) portion of Gag [27, 28], we hypothesized that the amino acid substitutions in the extracellular portion of gp41 may cause more A3G to remain outside the core, potentially mediated through a MA-NC interaction [29, 30]. To investigate this core exclusion mechanism, viral particles from SupT11-A3G cells were fractionated by ultracentrifugation through a 30 to 70% sucrose gradient with or without a top layer of Triton X-100. This fractionation method results in recovery of mature cores in fractions with a density of 1.24–1.28 g/ml [31], and intravirion localization of A3G was assayed by immunoblotting together with p24 Gag, RT, and Integrase (IN) which all colocalize to core fractions. In all instances, with or without detergent, A3G co-sedimented with established core components p24, RT, and IN (Fig 4A). Thus, the selected Env amino acid substitutions appear to have no detectable effect on the intravirion localization of A3G.

HIV-1 Env has a transmembrane domain and a cytoplasmic tail capable of signal transduction by activating TGF-β activated kinase 1 and NF-κB [28, 32]. Phosphorylation has also been implicated in negative regulation of A3G activity [33, 34]. These observations raised the possibility that Env adaptations may be leading to the inhibition of A3G activity by a signal transduction mechanism, potentially mediated through the MA-NC interaction mentioned in the previous paragraph. To test this possibility, viral particles produced from SupT11-A3G were purified and subjected to endogenous reverse transcription (ERT) *in vitro* [35], and the resulting viral cDNAs were analyzed by 3D-PCR and sequencing. In both analyses, significant levels of A3G-mediated G-to-A hypermutation were observed (Fig 4B and 4C and S8 Fig). The G-to-A mutation loads were approximately 2-fold lower than those in the Vif-null parental virus (Fig 4C), which did not account for the full resistance phenotype but suggested that the mechanism conferred by the adaptive Env changes may already be present in nascent virus particles (addressed further below).

### Env adaptations increase RT packaging, elevate reverse transcription levels, and prevent A3G-mediated hypermutation

**Fig 5:**
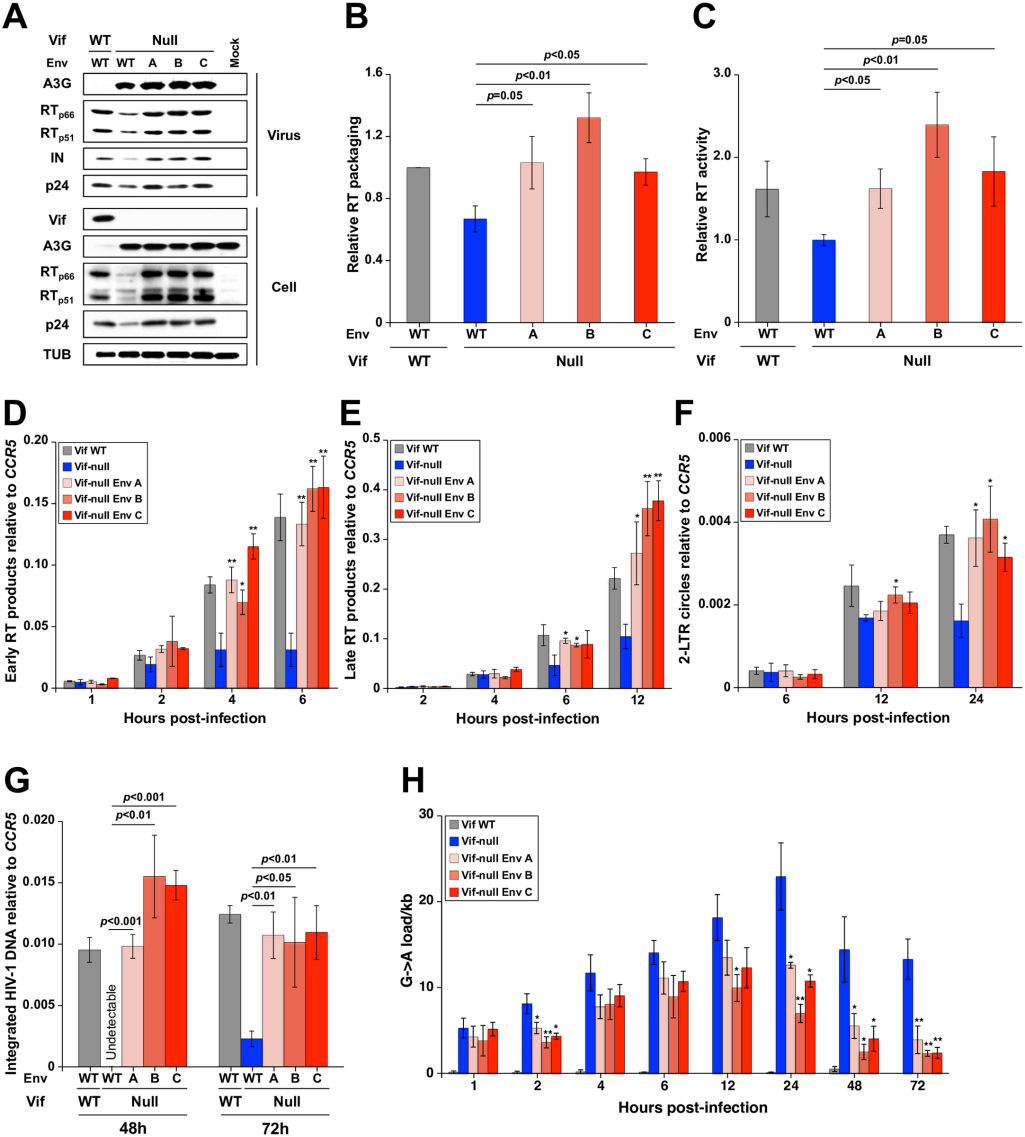
Env adaptations increase RT packaging, accelerate reverse transcription, and reduce G-to-A mutation levels. (A) Representative immunoblots of the indicated proteins in viral particles and producer cells from one experiment. (B) Relative RT packaging into viral particles produced in SupT11-A3G cells. RT packaging levels were quantified based on band intensity and normalized by each p24 of the virions (mean +/- SD of 3 biologically independent experiments). (C) Relative RT activity in viral particles produced in SupT11-A3G cells. RT activity was measured for each viral lysate normalized to p24 levels (mean +/- SD of 3 biologically independent experiments). (D to G) Kinetics of early RT, late RT, 2-LTR circle, and proviral DNA during infection of CEM-GFP cells using viruses originally produced in SupT11-A3G cells (mean +/- SD of 3 biologically independent experiments). (H) G-to-A mutation loads of high-fidelity, high-temperature *pol* amplicons from CEM-GFP cells infected with the indicated viruses (mean +/- SD of 3 biologically independent experiments with a minimum of 10 sequences or 5,640 bp per condition). Statistical comparisons for data in panels B-H were done using Student’s t test (*p*-values above each panel in comparison to Vif-null HIV-1; *: *p*<0.05, **: *p*<0.01, ***: *p*<0.001).

As shown above, A3G is correctly localized and enzymatically active in Vif-null Env variant viral particles. This finding strongly suggests the Env variants escape A3G restriction by an additional mechanism. Interestingly, immunoblot experiments with Env variant viral particles produced in SupT11-A3G cells revealed about 1.5 to 2-fold more encapsidated RT in comparison to the Vif-null parental virus (representative blot in Fig 5A with quantification of data from 3 independent experiments in Fig 5B). ELISA results corroborated this finding with purified adapted Env particles containing 1.5 to 2-fold more RT activity in comparison to the Vif-null parental virus (Fig 5C).

These results prompted us to ask whether higher levels of packaged RT in viruses from SupT11-A3G cells lead to corresponding increases in viral reverse transcripts, 2-LTR circles, and integrated DNA in CEM-GFP reporter cells. Established quantitative PCR (qPCR) assays [36, 37] were used to measure these virus replication products at multiple time points post-infection. For all three Env variants, strong increases in early RT product accumulation were evident, particularly 6 h post-infection where levels were 4- to 5-fold greater than Vif-null parental virus (Fig 5D). Corresponding increases were seen for accumulation of late RT products, 2-LTR circles, and integrated proviral DNAs (Fig 5E–5G). Notably, in the presence of A3G, the adapted Vif-null Env variants showed RT product accumulation kinetics similar to those of the Vif-proficient virus, whereas the RT kinetics of Vif-null HIV-1 with parental Env invariably lagged behind (consistent with a proposed model for A3G-dependent inhibition of reverse transcription [17, 38]). Similarly enhanced RT packaging and kinetics were evident with viruses produced in SupT11-vector cells, showing that these adapted Env phenotypes, though selected by A3G, can now manifest independent of this restriction factor (S9 Fig).

Given the increased RT kinetics resulting from each set of Env adaptations and the fact that A3G acts upon single-stranded DNA substrates, we asked if this mechanism might explain the lower levels of G-to-A mutation observed in viruses from SupT11-A3G cells (Fig 3B). This was addressed by sequencing the *pol* region at the sampling time points used above. As expected, G-to-A mutations were rare in cDNAs from Vif-proficient viruses throughout the entire time course (Fig 5H). In contrast, the *vif*-null parental virus showed high levels of G-to-A mutation at all time points with a peak of nearly 25 G-to-A mutations/kb at 24 h post-infection. Env adapted Vif-null viruses accumulated lower but still significant levels of G-to-A mutations at early time points consistent with results from endogenous reverse transcription experiments (compare data in Fig 5H versus Fig 4C). However, most interestingly, these hypermutation levels dissipated by 48 h and beyond to an average of less than 5 G-to-A mutations/kb (i.e., to sublethal levels consistent with single time point data in Fig 3B). Overall, these results indicated that the Env adapted particles have higher RT levels, which in turn enable faster rates of reverse transcription and fewer single-stranded DNA substrates for A3G to act upon.

### Elevated Gag-Pol packaging in A3G-adapted Env variant particles

**Fig 6:**
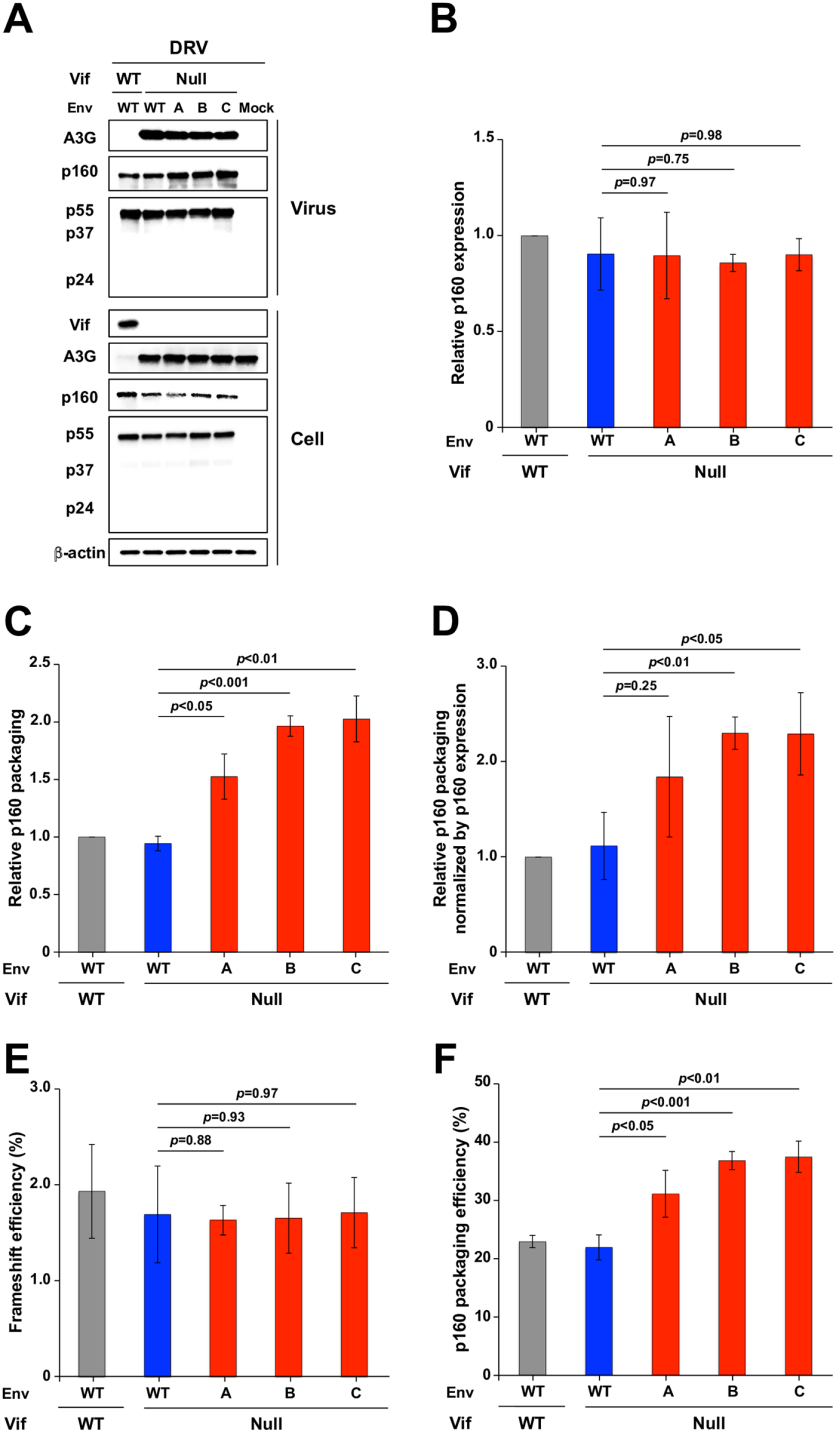
Env adaptations elevate levels of Gag-Pol packaging. (A) Immunoblot data from pseudo-single cycle assays of the indicated viruses produced in SupT11-A3G cells treated with the protease inhibitor DRV at 20 μM concentration. Immunoblots of the indicated proteins in viral particles and producer cells from one representative experiment of 3 biologically independent experiments. (B) p160 expression in SupT11-A3G cells infected with the indicated viruses. p160 expression levels were quantified by determining cellular band intensities, normalized to levels for WT virus, and then dividing by relative p55 levels (mean +/- SD of 3 biologically independent experiments). (C) p160 expression in the indicated viral particles produced from SupT11-A3G cells. p160 packaging levels were quantified by determining viral particle band intensities, normalizing to levels for WT virus, and then dividing by relative p55 levels (mean +/- SD of 3 biologically independent experiments). (D) p160/p55 ratios in viral particles relative to those in cells (values from panel C divided by those in panel B; mean +/- SD). (E) p55 to p160 ribosomal frameshift efficiency in SupT11-A3G cells infected by viruses with the indicated genotypes. p160 expression levels were quantified based on band intensity and divided by the sum of the p160 and p55 band intensities (mean +/- SD of 3 biologically independent experiments). (F) Efficiency of p160 packaging into viruses with the indicated genotypes produced in SupT11-A3G cells. p160 expression levels were quantified based on band intensity and divided by the sum of the p160 and p55 band intensities (mean +/- SD of 3 biologically independent experiments). Statistical comparisons for data in panels B-E were done using Student’s t test (*p*-values above each panel in comparison to data for Vif-null HIV-1).

To test the idea that Gag-Pol packaging is increased in A3G-adapted Env variants, a series of experiments was conducted with the protease inhibitor darunavir (DRV) to prevent processing of the 160 kDa Gag-Pol polyprotein (Fig 6). As above, VSV-G pseudotyped viral stocks were used at MOI 0.25 to infect SupT11-A3G cells. After allowing 6 h for infection to occur and well before new particles are produced, 20 μM DRV was added to each culture to allow the accumulation and accurate quantification of unprocessed p160 Gag-Pol and p55 Gag. Particle production was allowed to proceed an additional 42 h prior to harvesting cell free supernatants, virus concentration, and analysis by immunoblotting. As a negative control, a parallel reaction was treated with an equal volume of DMSO alone. In the presence of DRV, approximately 2-fold more Gag-Pol polyprotein (p160) was packaged into the adapted Vif-null particles in comparison to the Vif-null parental virus despite similar Gag-Pol expression levels in the DRV-treated cells (representative immunoblots in Fig 6A). This enhanced packaging phenotype was confirmed by quantification of the relative cellular levels of p160/p55, the relative viral levels of p160/p55, and the ratio of these two ratios (Fig 6B–6D, respectively). Moreover, detection of p160 and p55 on the same immunoblot with an anti-p24 monoclonal antibody enabled direct quantification of the rate of intracellular Gag-Pol to Gag ribosomal frameshifting, which was the same for all viral isolates (~2% Gag-Pol to Gag; Fig 6E). Most importantly, this approach also enabled direct quantification of the Gag-Pol to Gag ratios in virus particles, which were elevated 2-fold in the Env adapted molecular clones in comparison to controls (~40% versus 20%, respectively) (Fig 6F). These results further support the idea that enhanced Gag-Pol packaging is an essential feature of the Env-dependent A3G resistance mechanism.

### Overcoming A3G restriction requires virus production in the absence of syncytium formation

**Fig 7:**
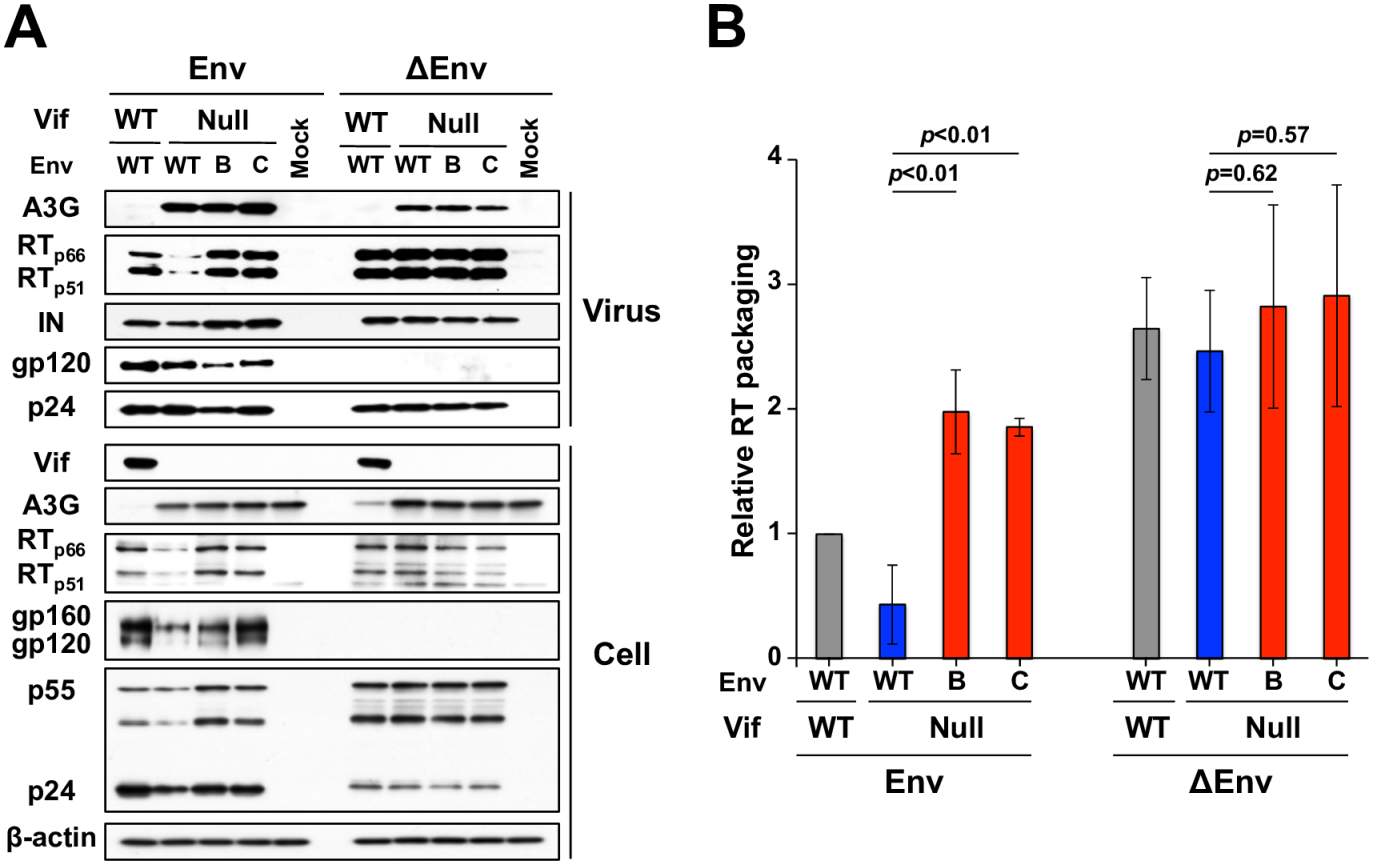
Env is a critical determinant of Gag-Pol packaging. (A) Immunoblot data from pseudo-single cycle assays of the indicated viruses with an intact *env* (left) or *env* deletion (right) produced in SupT11-A3G cells. Immunoblots for the indicated proteins in viral particles (top panels) and producer cells (bottom panels) from one experiment representative of 3 biologically independent experiments. (B) Quantification of RT (p66 and p51) packaging levels in the indicated viruses with and without Env produced in SupT11-A3G cells. Viral particle RT band intensities were normalized to levels for WT virus, and then divided by relative p24 levels (mean +/- SD of data from 3 biologically independent experiments).

A major prediction based on the results presented above, in Fig 6, and indeed also in S6 Fig, is that A3G restriction can be overcome by elevating RT amounts when viral particles are made in producer cells in which syncytium formation is rare because they express either a relatively less fusogenic Env or no Env at all. This prediction was tested by producing VSV-G pseudotyped viruses with and without Env using 293T cells, infecting the SupT11-A3G T cell line with an MOI of 0.25, and 48 h later analyzing key components of these cells and the resulting virus-containing supernatants. As above, the Env adapted, Vif-null viruses packaged 2-fold more RT (p66 and p51) in comparison to the Vif-null parent strain from which they were derived (representative immunoblots in Fig 7A and quantification in Fig 7B). However, Env-null particles, which were produced in SupT11 cells unable to undergo Env-mediated syncytium formation, showed no difference in RT packaging. Env-null viral particles also consistently showed the highest levels of RT packaging, though this difference was not statistically higher than levels observed in Env variants lacking Vif. Altogether, these data confirmed that levels of Gag-Pol and thus RT packaging are strongly influenced by Env and inversely correlated with fusogenicity of the producer SupT11-based T cell culture.

## Discussion

Proteasomal degradation is the major currently accepted mechanism used by HIV-1 Vif and related lentiviruses to counteract restriction by cellular A3 enzymes (reviewed by [3–5]). Here Vif-null HIV-1 was adapted to escape lethal restriction by A3G in order to probe for alternative mechanisms and learn more about the overall restriction mechanism. These studies revealed an unanticipated resistance mechanism mapping to the ectodomain of the gp41 subunit of Env (Fig 1). This mechanism is different than previously described work from our group [12, 13] and initially puzzling because it was not clear how amino acid changes on the outside of Env could confer resistance to A3G, which exerts restriction activities within capsid-encased viral cores. However, several observations combined to reveal multiple steps in a novel molecular mechanism. First, cell-cell fusion experiments showed that the adapted Env proteins form 2-fold fewer syncytia (Fig 2 and S4 and S5 Figs). Second, despite fully restrictive amounts of A3G being packaged, Vif-deficient viruses with adapted Env accumulate sublethal levels of G-to-A mutation, a result mirrored by reduced mutation accumulation in endogenous RT assays (Figs 3B, 4C and 5H). Third, a major advance in understanding the mechanism came from several experiments that showed 1.5- to 2-fold higher RT levels in adapted Env particles and a direct correspondence with enhanced reverse transcription kinetics and decreased levels of G-to-A hypermutation (Figs 5 and 7). Finally, experiments with the HIV-1 protease inhibitor DRV showed that, relative to the Vif-null parental virus, elevated levels of RT in the adapted Env variants are due to increased packaging of the Gag-Pol polyprotein (Fig 6). This differential Gag-Pol packaging capacity required viral particles to be produced under conditions that minimize syncytium formation (S6 Fig and Fig 7). These data combined to reveal an unanticipated homeostatic relationship between Env fusogenicity, Gag-Pol packaging, viral RT levels, and A3G restriction activity.

**Fig 8:**
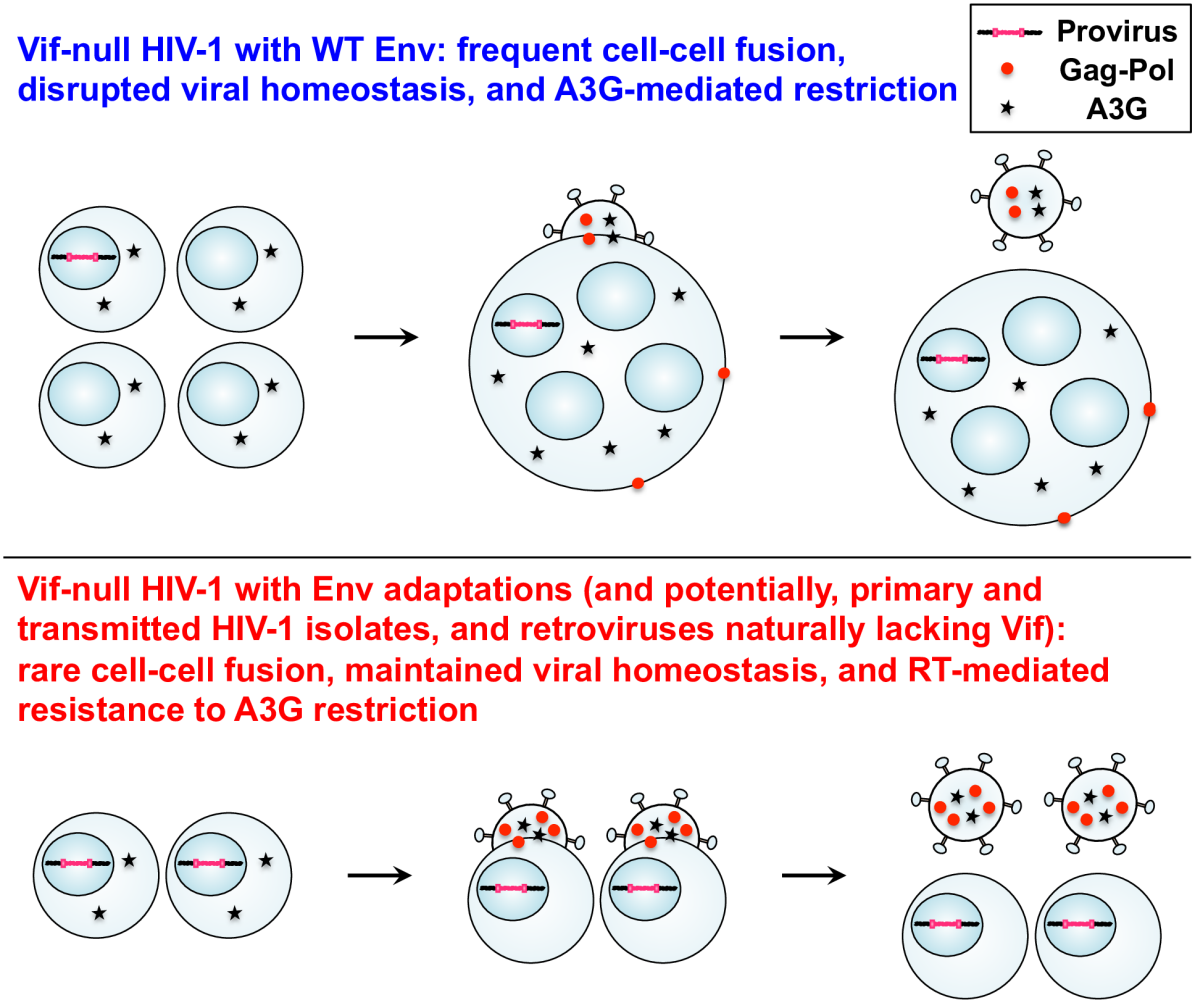
Model relating cell-cell fusogenicity, viral homeostasis, and A3G antiviral activity. The upper panel depicts a *vif*-null scenario with frequent syncytia and disrupted viral homeostasis characterized by less Gag-Pol packaging, slower reverse transcription, and a high susceptibility to A3G-mediated restriction. The lower panel depicts an adapted virus scenario with few syncytia and restored viral homeostasis including more Gag-Pol packaging, higher rates of reverse transcription, and resistance to A3G-mediated restriction. Individual cells are depicted with one nucleus, and fused cells with four nuclei. For simplicity, individual cells are shown producing particles with 4 units of RT and 2 units of A3G, whereas the syncytium is shown producing particles with 50% less RT (2 units) with the same amount of A3G (2 units). See text for additional explanation.

Our results can be summarized in a model in which fusogenicity is a major factor governing overall viral homeostasis (Fig 8). HIV-1 isolates with a (relatively) high fusogenic potential will cause infected host cells to form multinucleated syncytia, which increases cellular volume, decreases ratios of viral to cellular components, and causes an overall disruption in viral homeostasis. For instance, viral components such as Gag-Pol become diluted, whereas cellular components such as A3G likely remain stoichiometric with the number of fused cells. Thus, Vif-null viral particles assembling from wild-type Env-fused cells will have less Gag-Pol polyprotein, slower rates of reverse transcription, normal levels of cellular factors including A3G, and normal amounts of Gag (or particles would not be available for experimentation; top schematic in Fig 8). Slower rates of reverse transcription lead to more single-stranded cDNA replication intermediates, increased opportunities for A3G-catalyzed deamination, and high levels of G-to-A hypermutation and restriction. In contrast, the adapted Env variants described here have less fusogenicity, which restores viral homeostasis by enabling more Gag-Pol packaging, higher rates of reverse transcription, and protection from lethal levels of A3G-mediated restriction (bottom schematic in Fig 8). As interesting side observations, our studies also indicated that Vif has additional functions beyond APOBEC degradation in virus protein production in producer cells (e.g., Fig 3A) and, perhaps related, in virus transmission and/or preventing fusion (Fig 2 and S4 Fig). We speculate that these effects may be due to Vif’s documented role in promoting cell cycle arrest [39] and/or in degrading regulatory subunits of the cellular PP2A phosphatase complex [40], either of which may also contribute to overall viral homeostasis.

Reverse transcription is the defining feature of retrovirus and retrotransposon replication and, with knowledge of the results presented here, it becomes more apparent that regulation of this process will dramatically impact the access of A3G to viral first-strand cDNA replication intermediates. Many factors govern the ratio of structural proteins (e.g., Gag) to enzymes (e.g., RT) packaged into viral particles including translational recoding processes required to produce Gag-Pol polyprotein (ribosomal frameshifting or nonsense codon read-through [41, 42]). It is surprising, as shown here, that HIV-1 Env fusogenicity and overall viral homeostasis is also a major factor in determining the amount of Gag-Pol and therefore also the amount of RT that ultimately becomes encapsidated. In further support of this homeostatic model as well as suggesting an additional layer of viral counterdefense, a recent study from the Malim group documented an inhibitory interaction between HIV-1 RT and A3G [43]. Thus, elevated RT levels may have dual benefit for the virus by both accelerating RT kinetics and blocking A3G from accessing viral cDNA. Interestingly, the resistance mechanism described here does not protect Vif-null HIV-1 from restrictive levels of A3F (Fig 1D, S2 Fig and S1 and S2 Tables). These results support the notion, inferred previously in complementary studies [12–14, 17], that A3F and A3G use at least partly distinct mechanisms to restrict HIV-1. For instance, the antiviral effects of A3F have been attributable to a greater extent to deaminase-independent mechanisms [17, 44], A3F has 10-fold higher affinity than A3G for single-stranded DNA [45], and A3F (but not A3G) can be used to microscopically track single viral particles from the fusion stage at the cell membrane all the way to the pre-integration stage within the nuclear compartment [46]. Thus, it will be interesting in future studies to ask whether RT might also directly interfere with A3F restriction activity and to define additional adaptations and mechanisms that are likely necessary to overcome restriction by A3F.

Clinical HIV-1 isolates can be classified broadly by co-receptor usage. Most transmitting HIV-1 isolates as well as viral isolates recovered during the early stages of infection utilize CCR5, whereas a subset of isolates found at later stages of infection such as during the development of AIDS utilize CXCR4. CCR5 isolates are less capable of syncytium formation than CXCR4 isolates in T cell cultures, and this is primarily due to low levels of CCR5 expression on many T cell subsets [47–49]. It is therefore noteworthy that the Env adaptations described here in the C-terminal heptad repeat region of gp41 occur in the vicinity of adaptive changes that enable virus replication in cells expressing low levels of CCR5 [50]. It is further noteworthy that the Env adaptations described here in the C-terminal heptad repeat region of gp41 (T626M and H643Y) make the protein more similar to Env observed in the majority of transmitted/founder and chronic HIV-1 isolates [15]. Thus, the novel Env/RT-mediated mechanism described here may help to protect the genetic integrity of the virus during transmission and/or to maintain the overall homeostasis of the infection process. Such a parallel or alternative mechanism may be advantageous in the earliest stages of virus production by a newly infected host cell when Vif may not have had sufficient time to purge cells of potentially harmful levels of A3G. This mechanism may also act as protection for viruses with compromised Vif function (as may be selected by T cell responses), which would allow time for additional virus evolution and restoration of Vif function.

The Env- and RT-mediated mechanism described here may also have broader relevance because reverse-transcribing parasites (retroviruses and retrotransposons) are ubiquitous and the number of known A3 counteraction mechanisms is still quite limited. First, the Vif-dependent A3 counteraction mechanism is unique to lentiviruses (reviewed by [4]). Second, spumaviruses appear to antagonize cellular A3s through a protein called Bet [51–54]. Last, human T-lymphotropic virus-1 (HTLV-1) appears to exclude A3 enzymes from particles (i.e., a passive avoidance mechanism [55]). Therefore, alpha-retroviruses such as Rous sarcoma virus (RSV), gamma-retroviruses such as murine leukemia virus (MLV), and/or beta-retroviruses such as mouse mammary tumor virus (MMTV) among many other examples may avert restriction by cellular A3 enzymes simply by having faster overall rates of reverse transcription that, as shown here, are able to effectively shield single-stranded cDNA intermediates from lethal mutation by A3G. In support of this general mechanism, MMTV is partially resistant to restriction by murine A3 [56], and this property can be changed in a predictable manner by mutating RT or RNase H and thereby altering the accessibility of viral single-stranded cDNA to cellular APOBEC enzymes (B. Hagen, M. Kraase, and S. Indik, 2016, Cold Spring Harbor Laboratory (CSHL) Meeting on Retroviruses [abstract #109]).

Arguably the most intriguing finding here is that fusogenicity is an adaptable component of viral homeostasis that, with even modest change, can alter the ability of HIV-1 to overcome catastrophic damage by a potent virus restriction factor. If so, we predict that changes in Env fusogenicity might also be able to overcome restrictions beyond that imposed by A3G. Indeed, feline immunodeficiency virus was shown to evolve fusion-reducing changes in Env in order to resist deleterious effects of a dominant negative TSG101 fragment [57]. HIV-1 also adapted in an analogous Env-dependent manner to overcome a defect in the ALIX-binding motif of p6 (R. Van Duyne, L. Kuo, K. Fuji, and E. Freed, 2015, CSHL Meeting on Retroviruses [abstract #232]). Finally, Env mutations that lead to decreased fusogenicity have even been shown to allow HIV-1 to overcome inhibition by the integrase inhibitor dolutegravir (R. Van Duyne, L. Kuo, K. Fuji, and E. Freed, 2017, CSHL Retroviruses Meeting on Retroviruses [abstract #6]). Taken together, these findings point towards an emerging and potentially general theme in virus pathogenesis: obstacles to virus replication may be overcome by adaptations in Env that modulate fusogenicity and syncytium formation. Thus, we propose a new paradigm in which viral homeostasis is maintained through fine-tuning essential viral functions to offset perturbations to other functions. This paradigm may constitute a general mechanism used by HIV-1 and other viral pathogens to overcome obstacles to virus replication.

## Materials and methods

### Key reagents

293T (CRL-3216) and HeLa (CCL-2) cells were obtained from American Type Culture Collection. CEM-SS (#776) [58], CEM-GFP (#3655) [59], CEM.NKR-CCR5-Luc (CEM-luc; #5198) [60] and TZM-bl (#8129) [61] cells were obtained from the NIH AIDS Reagent program. SupT11 stably expressing a vector control, untagged A3F, or untagged A3G [62], CEM2n [8], and CEM-SS stably expressing a vector control, untagged A3F, or untagged A3G [12] were previously created and validated by the Harris lab. Adherent cells were maintained in DMEM supplemented with 10% fetal bovine serum (FBS) and 0.5% penicillin/streptomycin (P/S). All T cell lines used in this study were cultured in RPMI with 10% FBS and 0.5% P/S.

pcDNA4/TO-3xFlag has been described [63]. An *A3G* cDNA was amplified with RSH8373/8374, cut with *Eco*RV and *Not*I, and inserted into similarly digested pcDNA4/TO-3xFlag. Expression plasmids Rluc8-DSP^1−7^ and Rluc8-DSP^8−11^ were provided by Dr. Zene Matsuda (University of Tokyo [64]).

Anti-A3F (#11474), A3G (#10201), gp120 (b12, #2640), gp41 (2F5, #1475), p24 (183-H12-5C, #1513 and AG3.0, #4121), Vif (319, #6459) and RT (8C4, #7373, and #6195) antibodies, DRV (#11447) EFV (#4624) and C34 (#9824) were obtained from the NIH AIDS Reagent program. Anti-Flag (M2, Sigma), beta-actin (4970S, Cell Signaling), TUB (MMS-407R, Covance), gp120 (20-HG81, Fitzgerald), and IN (NBP1-00584, Novus) antibodies were purchased.

S3 Table lists the DNA oligonucleotides used in this study, which correspond to RSH collection numbers.

### Virus constructions

Vif-proficient (GenBank EU541617) and Vif-deficient (X26 and X27) HIV-1 IIIB C200 proviral expression constructs have been reported [12, 14]. A 230 bp *vif* deletion was made by overlapping PCR using primer sets (RSH1451/4068 and RSH4069/1452), and cloned into the Vif-deficient proviral construct at *Swa*I and *Sal*I sites. To construct proviral DNA expression plasmids encoding substitutions in *env*, the *env* gene was divided into 4 pieces based on unique restriction enzyme sites, and each fragment was cloned into pJET1.2/blunt cloning vector (Thermo Scientific) by amplifying with the following primer sets: RSH7808/7809 (*Sal*I/*Nde*I), RSH7813/7814 (*Nde*I/*Nhe*I), RSH7817/7818 (*Nhe*I/*Bam*HI), and RSH8086/8087 (*Bam*HI/*Xho*I). Substitutions were introduced by site-directed mutagenesis using primers, RSH7815/7816 for P79L, RSH7819/7820 for M687I, and RSH8088/8089 for V822I. Other substitutions were constructed by subcloning DNA fragments including mutations of interest in relevant gp120 or gp41 regions described above. Finally, DNA fragments containing the substitutions in *env* were inserted into the *vif*-null proviral expression vector using unique restriction enzyme sites. To generate *Δenv* proviral plasmids, DNA fragments amplified by RSH7808/7814 were subcloned into pJET1.2/blunt cloning vector, digested with *Nde*I, and treated with DNA blunting enzyme (Thermo Scientific) to insert 2 nucleotides.

### Adaptation experiments

SupT11-A3G was used in the first round and CEM2n in the second round of step-wise virus adaptations based on prior reports [10, 12, 14] (Fig 1A). Vif-null HIV-1 IIIB was used to infect 1.5x10^5^ permissive cells (SupT11-vector) in a total volume of 1 ml in one well of a 24 well plate at a multiplicity of infection (MOI) of 0.05. Infected cells were split and the media were replenished at 4 days post-infection to prevent overgrowth. Cell free supernatants were used to monitor virus replication every 4–8 days by infecting 2.5x10^4^ CEM-GFP reporter cells and, 3 days later, analyzing the percentage of GFP-positive cells by flow cytometry. On day 8 post-infection, 250 μl of cell-free supernatant was transferred to a mixed culture of nonpermissive and permissive culture of 1.5x10^5^ T cells (SupT11-A3G and SupT11-vector or CEM2n and CEM-SS). The step-wise cultures were done in order of 25:75, 50:50, 75:25 and 100:0 ratios of nonpermissive/permissive cells. Adapted viruses that emerged from this protocol were purified by three additional rounds of passage under fully nonpermissive conditions (0.01 MOI with fresh SupT11-A3G or CEM2n). Finally, overlapping primer sets and high-fidelity PCR were used to recover proviral DNA fragments for cloning into pJET1.2/blunt vector and DNA sequencing (5’LTR to 3’LTR: RSH2456/1431, RSH1432/1433, RSH1434/1435, RSH1451/1454, RSH1440/1441, RSH1442/1443, RSH1444/1445, and RSH1649/7418). 8–12 clones were sequenced for each amplicon and nucleotide changes were defined as fixed mutations only when they occurred in at least 75% of sequences.

### Immunoblots

Cells were lysed in 25 mM HEPES (pH 7.4), 150 mM NaCl, 10% glycerol, and 1% NP40 and virions were dissolved after pelleting in 2xSDS sample buffer [100 mM Tris-HCl (pH 6.8), 4% SDS, 12% 2-mercaptethanol, 20% glycerol, 0.05% bromophenol blue]. Proteins in cell and viral lysates were fractionated by SDS PAGE, transferred to PVDF membrane (Millipore), and blocked with 4% milk in PBS containing 0.1% Tween 20. In all experiments, viral lysates were normalized by p24 immunoblot and/or ELISA levels and re-analyzed through additional rounds of immunoblotting. Subsequently, membranes were incubated with primary antibodies, horseradish peroxidase (HRP)-conjugated secondary antibodies, developed using HyGlo chemiluminescent HRP detection reagent (Denville Scientific) and exposed to film. Band intensity was analyzed by using Image J software.

### Virus infectivity experiments

HIV-1 spreading infection assays were performed as described [14]. Viruses were produced by transfection of 3.0 μg of proviral expression construct using TransIT-LT1 reagent (Mirus Bio) into 293T cells (3.0x10^6^). 48 h later, virus-containing supernatants were filtered by 0.45 μm filters (Millipore) and used to infect into 2.5x10^4^ CEM-GFP reporter cells for MOI determinations. Spreading infections were initiated at a MOI of 0.01 for 1.5x10^5^ target cells and infectivity was monitored every 2 or 4 days by the CEM-GFP system.

Single cycle experiments were initiated by transfecting 2.5x10^4^ 293T cells with 1.0 μg of proviral expression construct and 20 ng vector control, 4 ng pcDNA4/TO-A3G-3xFlag supplemented with 16 ng vector control, or 20 ng pcDNA4/TO-A3G-3xFlag using TransIT-LT1 reagent. 48 h later, virus-containing supernatants were filtered and used to infect into 2.5x10^4^ CEM-GFP cells for titrations. Pseudo-single cycle assays with T cell lines were done as described [12]. VSV-G pseudotyped viruses were generated by transfecting 2.4 μg of proviral DNA construct and 0.6 μg of VSV-G expression vector into 293T cells. At 48 h post-transfection, supernatants were harvested, filtered, and titered using the CEM-GFP system. 10^5^ target T cells were infected with a MOI of 0.25 and then washed with PBS after 6 h of the infection. 42 h later, supernatants were collected, filtered, and used to infect into CEM-GFP. 20 μM DRV was added 6 h post-infection and cultures were continued for an additional 42 h to obtain sufficient viruses for immunoblotting.

### Hypermutation analyses

Infected CEM-GFP reporter cells were used to generate genomic DNA (Puregene). Following *Dpn*I digestion, the viral *pol* region was amplified by nested PCR with RSH4196/4197 as outer primers (876 bp) and RSH4205/4206 as inner primers (564 bp) and then the amplified products were subjected to pJET cloning and DNA sequencing.

In some experiments, 3D-PCR was used to provide a semi-quantitative assessment of G-to-A hypermutation [7, 10, 65]. This differential DNA denaturation (3D) technique is based on the fact that any given DNA fragment will have a characteristic melting point, and decreasing the number of G/C base pairs in favor of A/T by hypermutation will decrease the hydrogen-bonding potential and enable lower temperature PCR amplification. An 876 bp *pol* fragment was amplified from proviral DNA using RSH4196/4197 and the relative amount of this amplicon was quantified by qPCR (LightCycler 480, Roche). Normalized amounts were then used for a second PCR reaction using RSH4205/4206 to generate a 564 bp product with a denaturation temperature ranging from 77 to 85°C. PCR products were run on agarose gel and detected by ethidium bromide staining.

### Sucrose density gradient centrifugation

Viral stocks were prepared as above, filtered to remove cellular debris, and concentrated by centrifugation (26,200 x *g*, 4°C, 2 h) through a 20% sucrose cushion. The resulting viral pellets were suspended in STE (10 mM Tris-HCl pH7.4, 100 mM NaCl, 1 mM EDTA) and subjected to SW41 ultracentrifugation (100,000 x *g*, 4°C, 16 h; Beckman) through a linear 30 to 70% (w/v) sucrose gradient including a 1% Triton X-100 layer at the top, as described [31, 66]. After centrifugation, eleven 1 ml fractions were collected from the top of the gradient and each fraction was diluted into STE buffer to pellet down proteins. Individual fractions were analyzed by immunoblotting using antibodies listed above.

### ERT assays

ERT assays were performed as described [35, 67]. Viral supernatants produced from pseudo-single cycle assays were purified by a centrifugation through a 20% sucrose cushion. The resulting viral pellets were suspended in PBS with 15 μg/ml melittin (Sigma), 2.5 mM MgCl_2_, and 1 mM dNTPs and incubated 37°C overnight. The resulting viral cDNAs were isolated using Puregene reagents with 100 μg/ml of salmon sperm DNA. After treatment with *Dpn*I, the *pol* region of the isolated viral cDNAs was amplified, cloned into pJET1.2/blunt cloning vector and sequenced. The G-to-A mutation load per kb from four independent replicate experiments was analyzed by one-way ANOVA using GraphPad Prism 6, and Fisher’s LSD test was used to determine statistical significance (*p*<0.05).

### RT assays

Viral stocks were prepared as above, filtered, and concentrated by centrifugation. The resulting viral particles were normalized by p24 ELISA (ZeptoMetrix) and RT activity was measured by an ELISA-based reverse transcriptase assay (Roche).

### qPCR

Equivalent amounts of cell-free virus produced in SupT11-vector or -A3G cells and normalized by p24 levels were used to infect 10^6^ CEM-GFP cells. At 1, 2, 4, 6, 12, 24, 48, and 72 h postinfection, viral cDNAs were isolated using Puregene reagents and digested with *Dpn*I. Primers and probes for total RT products (RSH9972/9973/9974), late RT products (RSH9975/9976/9977), 2-LTR circles (RSH9978/9979/9980), and integrated viral DNAs (RSH9978/9981/9980) were designed based on the prior reports [36, 37]. 100 ng template DNA was subjected to qPCR using a LightCycler 480. Early RT products were calculated by subtracting late RT products from total RT products. All products were normalized to the cellular *CCR5* gene (RSH9982/9983/9984).

### Transmission/Fusion assays

To simultaneously measure virus transmission and virus-induced cell-cell fusion in T cell co-cultures, we used our previously reported assay [19] with minor modifications. 1.5x10^6^ 293T cells were cotransfected by calcium phosphate precipitation (Invitrogen) with 8.5 μg of the respective proviral DNA expression plasmids and 1.5 μg of a VSV-G expression plasmid, and the medium was refreshed after 24 h. 36 h later, supernatants were filtered and used to infect CEM-SS cells at a MOI of 0.3. After washing, cells were incubated for 48 h, counted and resuspended in fresh medium. Then, CEM-luc target cells were co-cultured with infected cells at a 2:1 ratio (target:infected) in 1.2 ml total medium in wells of a 24-well plate. DMSO, EFV (2.5 μM final), or EFV and C34 (500 nM final) were added to target cells. After 24 h, 600 μl supernatant was removed, and 600 μl fresh medium was added containing both EFV and C34 at the above concentrations to fully block any further transmission or cell-cell fusion. After allowing a further 24 h for infected CEM-luc target cells or CEM-SS/CEM-luc syncytia to express the luciferase reporter gene (Fig 2A), cells were harvested and lysed in 100 μl cold lysis buffer (Promega) containing 1% protease inhibitor cocktail (Promega). Intracellular luciferase activity was measured by a Synergy 2 monochromator-based multimode microplate reader (BioTek). At the time of initiation of co-culture, 5.0x10^5^ infected cells from each condition were labeled with LIVE/DEAD Fixable Near-IR viability stain (Molecular Probes, 1:500 dilution) and fixed in 2% PFA/PBS. Cells were then treated with blocking/permeabilization buffer (B/P; PBS/1% BSA/0.2% Triton X-100) containing an anti-p24 mouse monoclonal antibody (AG3.0, 1 μg/ml) and incubated with a donkey anti-mouse-Alexa488 (Molecular Probes, 1:500) in B/P buffer. Cells were analyzed by flow cytometry on a BD LSR II cytometer using 488 nm and 633 nm excitation lasers and FITC and APC-Cy7 emission filter cubes.

Virus transmission was taken to be the portion of the signal sensitive to EFV, calculated by subtracting the relative light units of wells treated with EFV (for the first 24 h) from wells treated with DMSO vehicle (for the first 24 h). Cell-cell fusion was taken to be the residual signal after EFV inhibition, and was confirmed to be fusion-dependent signal by further inhibiting with C34, which consistently eliminated all luciferase signals to background levels (S3 Fig). For each experiment, the two technical replicates were averaged, these values were compared across four independent biological replicates by one-way ANOVA using GraphPad Prism 6, and *p*-values (Fisher’s LSD test) were calculated with significance indicated at *p*<0.05.

### Cell-cell fusion assays

An assay to measure HIV-induced cell-cell fusion was performed as described [64, 68]. HeLa cells were transfected with a proviral plasmid (or Env-deleted virus as a negative control) and a plasmid for expression of the N-terminal portion of a Renilla luciferase 8 (Rluc8)-GFP fusion protein (Rluc8-DSP^1−7^). TZM-bl cells serving as targets were in parallel transfected with the C-terminal portion of the DSP (Rluc8-DSP^8−11^). HeLa cells were re-plated to a 12-well format in quadruplicate for either fusion assays or surface Env level measurement. Transfected target cells were detached and overlayed on HeLa cells at roughly a 1:1 ratio. Upon cell-cell fusion, the two portions of the dual split protein assemble within syncytia, resulting in GFP fluorescence (see S4 Fig). After 4 h of co-culture, cells were fixed, permeabilized, and stained with an anti-p24 antibody (AG3.0) overnight. Finally, cells were stained with an anti-mouse-Alexa647 secondary antibody (Molecular Probes) and analyzed by flow cytometry. In parallel, to evaluate surface Env levels in the same HeLa cells, cells were harvested and surface-stained with a human anti-gp120 monoclonal antibody (b12) at 4°C. Then, cells were fixed, permeabilized, and stained with an anti-p24 monoclonal antibody. Finally, cells were stained with secondary antibodies, and analyzed by flow cytometry. For each experiment, the two technical replicates were averaged, these values were compared across three independent biological replicates by one-way ANOVA using GraphPad Prism 6, and *p*-values (Fisher’s LSD test) were calculated with significance indicated at *p*<0.05.

## Supporting information

S1 Fig Adaptation of Vif-null HIV-1 isolates to restrictive A3G levels in SupT11 cells. (A) Representative spreading infection data for A3G resistant isolates in the indicated SupT11 derivatives. Isolate A substitutions: MA V35I, Pro L33I, Pro M36I, RT R172K, RT L210W, Vpr Q11X, Rev S8N, gp120 A58V, gp120 A60T, gp41 H643Y, gp41 M687I, gp41 V822I, Nef R19K, Nef G67S, Nef E179K; Isolate B substitutions: CA E213D, NC R29K, RT T165I, RT R211K, Vpr W18X, Vpr I70L, gp120 P79L, gp120 S143N, gp120 M426L, gp120 Q442P, gp120 G464E, gp41 S640N, gp41 H643Y, gp41 M687I, gp41 S762N, Nef G12R, Nef A27V, Nef R35Q, Nef Q73X, Nef D186N; Isolate C substitutions: MA V35I, NC M46I, Pro M36I, Pro P79S, RT V179A, Vif N19D, Vpr Q11X, Vpr A59V, Vpr R62K, Vpr Q65X, Rev Q36R, Rev G93E, gp120 V38I, gp120 T278M, gp120 G410E, gp41 T626M, gp41 K655M, gp41 M687I, gp41 R729G, gp41 G786R, gp41 L851X, Nef R19K, Nef D186N. (B) An image of the ethidium bromide-stained agarose gel containing *vif-vpr* products (with *vif*: 2391 bp or without *vif*: 2161 bp) of the indicated A3G resistant isolates. Proviral DNAs were recovered from CEM-GFP cells infected with the resistant isolates produced in CEM2n cells and amplified with a primer set RSH1451/1454. Proviral plasmids (pIIIB Vif WT and Vif-null) and H_2_O are controls. (C) Mutation matrices derived from sequence analysis of each A3G resistant viral isolate.
(PDF)

S2 Fig Spreading infection phenotypes of Vif-null Env variant molecular clones. (A) Representative spreading infection assays in CEM-SS stably expressing a vector control or A3G (2 independent clones). (B) Immunoblots of A3G and TUB in the indicated cell lines. (C) Representative spreading infection assays in CEM-SS stably expressing a vector control or A3F (2 independent clones). (D) Immunoblots of A3F and TUB in the indicated cell lines.
(PDF)

S3 Fig EFV-resistant luciferase signal is completely eliminated by fusion inhibitor peptide C34. Total luminescence signals of Vif-proficient, WT Env virus treated with DMSO, EFV or EFV plus C34. Each signal is normalized to Gag expression, and reported as relative to the DMSO treatment. The fusion-dependent signal (EFV) falls to background level (mean; <-1 x 10^−4^) by the additional C34 treatment. Each histogram bar represents the mean +/- SEM of 4 biologically independent experiments.
(PDF)

S4 Fig Env variants exhibit reduced syncytium formation. (A) Cell-cell fusion assay schematic showing that GFP fluorescence only occurs upon reconstitution of the split GFP fragments in the same cytosol after fusion. Assay based on original studies [62, 66]. (B) Surface Env levels of HeLa cells transfected with the indicated viral constructs (flow cytometry data normalized to Vif-proficient condition for comparison). (C) Relative fusion efficiency of HeLa cells expressing the indicated HIV-1 constructs with TZM-bl target cells. Each experiment in panels B-C was done in triplicate and the average +/- SEM is shown for each condition. Statistical comparisons were done using a one-way ANOVA and Fisher’s LSD test (*p*-values above each panel).
(PDF)

S5 Fig Env mutations also reduce syncytium formation in the Vif-proficient context. (A) Total luminescence normalized to Gag expression and reported relative to the Vif-proficient virus. Each histogram bar represents the mean +/- SEM of the normalized data from 3 biologically independent experiments (*p*-values above each panel from one-way ANOVA and Fisher’s LSD test). (B) Luminescence signal attributable to cell-cell fusion or virus transmission for the indicated viruses. Cell-cell fusion events are quantified as the fraction of total luciferase signal that is resistant to EFV-treatment, and virus transmission events are quantified by subtracting the cell-cell fusion signal from the total luminescence signal. Each histogram bar represents the mean +/- SEM of 3 biologically independent experiments (*p*-values above each panel from one-way ANOVA and Fisher’s LSD test).
(PDF)

S6 Fig Env adaptations do not confer resistance to A3G in adherent 293T cells. A representative single-cycle assay in 293T cells. The histogram reports mean +/- SD infectivity of 3 technically independent experiments. The immunoblots below show the indicated proteins in viral particles and cell extracts.
(PDF)

S7 Fig Env adaptations protect Vif-null HIV-1 from A3G mutagenesis. (A) Representative viral mutation plots from 1 of 3 independent experiments. The indicated viruses were produced in SupT11-A3G cells and used to infect CEM-GFP cells, from which the *pol* region was amplified by high-fidelity PCR, cloned, and sequenced. (B) Actual distribution of G-to-A mutations in the indicated dinucleotide contexts in 10 independent 564 bp *pol* region DNA sequences from panel A.
(PDF)

S8 Fig Endogenous reverse transcription data. (A) Representative viral mutation plots from 1 of 4 independent ERT experiments. The *pol* region was amplified by high-fidelity PCR, cloned, and sequenced. (B) Actual distribution of G-to-A mutations in the indicated dinucleotide contexts in 10 independent 564 bp *pol* region DNA sequences from panel A.
(PDF)

S9 Fig RT packaging and RT kinetics of Env variants produced in SupT11-vector cells. (A) Immunoblots of the indicated proteins in viral particles and SupT11-vector producer cells from one representative experiment. (B) Relative RT packaging into viral particles produced in SupT11-vector cells. RT packaging levels were quantified based on band intensity and normalized by each p24 of the virions. Each data point is the average +/- SD of biological triplicates. (C) Relative RT activity in viral particles produced in SupT11-vector cells. RT activity in each viral lysate normalized for p24 contents was measured. Each data point is the average +/- SD of biological triplicates. (D-G) Kinetics of early RT, late RT, 2-LTR circle, and proviral DNA during infection of CEM-GFP cells using viruses originally produced in SupT11-vector cells (mean +/- SD of 3 biologically independent experiments). Statistical comparisons were done using Student’s t test (*p*-values above each panel in comparison to Vif-null HIV-1; *: *p*<0.05, **: *p*<0.01, ***: *p*<0.001).
(PDF)

S1 Table Summary of SupT11 spreading infection data. (PDF)

S2 Table Summary of CEM-SS spreading infection data. (PDF)

S3 Table Oligonucleotide sequences. (PDF)
