# Supplementary material for: HIV-1 adaptation studies reveal a novel Env-mediated homeostasis mechanism for evading lethal hypermutation by APOBEC3G
Source: PLoS Pathog. 2018 Apr 20;14(4):e1007010. doi: 10.1371/journal.ppat.1007010 (PMC5931688; doi:10.1371/journal.ppat.1007010)
Supplement: S1 Fig — (A) Representative spreading infection data for A3G resistant isolates in the indicated SupT11 derivatives. Isolate A substitutions: MA V35I, Pro L33I, Pro M36I, RT R172K, RT L210W, Vpr Q11X, Rev S8N, gp120 A58V, gp120 A60T, gp41 H643Y, gp41 M687I, gp41 V822I, Nef R19K, Nef G67S, Nef E179K; Isolate B substitutions: CA E213D, NC R29K, RT T165I, RT R211K, Vpr W18X, Vpr I70L, gp120 P79L, gp120 S143N, gp120 M426L, gp120 Q442P, gp120 G464E, gp41 S640N, gp41 H643Y, gp41 M687I, gp41 S762N, Nef G12R, Nef A27V, Nef R35Q, Nef Q73X, Nef D186N; Isolate C substitutions: MA V35I, NC M46I, Pro M36I, Pro P79S, RT V179A, Vif N19D, Vpr Q11X, Vpr A59V, Vpr R62K, Vpr Q65X, Rev Q36R, Rev G93E, gp120 V38I, gp120 T278M, gp120 G410E, gp41 T626M, gp41 K655M, gp41 M687I, gp41 R729G, gp41 G786R, gp41 L851X, Nef R19K, Nef D186N. (B) An image of the ethidium bromide-stained agarose gel containing vif-vpr products (with vif: 2391 bp or without vif: 2161 bp) of the indicated A3G resistant isolates. Proviral DNAs were recovered from CEM-GFP cells infected with the resistant isolates produced in CEM2n cells and amplified with a primer set RSH1451/1454. Proviral plasmids (pIIIB Vif WT and Vif-null) and H2O are controls. (C) Mutation matrices derived from sequence analysis of each A3G resistant viral isolate. (PDF) [file ppat.1007010.s001.pdf]

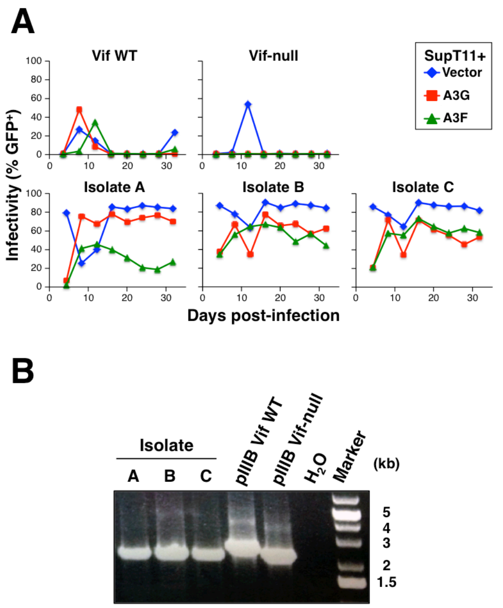

C

Isolate A (From / To)

To \ From	A	C	G	T
A		4	121	1
C	1		1	2
G	5	0		2
T	0	7	3	

GA; 23%, GG; 69%
GT; 5%, GC; 3%
35.5 kb sequenced
3.4 G→A mutations/kb

Isolate B (From / To)

To \ From	A	C	G	T
A		0	122	1
C	5		0	3
G	7	2		0
T	1	14	1	

GA; 30%, GG; 61%
GT; 3%, GC; 6%
35.5 kb sequenced
3.4 G→A mutations/kb

Isolate C (From / To)

To \ From	A	C	G	T
A		1	115	2
C	0		1	5
G	5	2		1
T	1	15	2	

GA; 30%, GG; 58%
GT; 10%, GC; 3%
35.5 kb sequenced
3.2 G→A mutations/kb

S1 Fig. Adaptation of Vif-null HIV-1 isolates to restrictive A3G levels in SupT11 cells. (A) Representative spreading infection data for A3G resistant isolates in the indicated SupT11 derivatives. Isolate A substitutions: MA V35I, Pro L33I, Pro M36I, RT R172K, RT L210W, Vpr Q11X, Rev S8N, gp120 A58V, gp120 A60T, gp41 H643Y, gp41 M687I, gp41 V822I, Nef R19K, Nef G67S, Nef E179K; Isolate B substitutions: CA E213D, NC R29K, RT T165I, RT R211K, Vpr W18X, Vpr I70L, gp120 P79L, gp120 S143N, gp120 M426L, gp120 Q442P, gp120 G464E, gp41 S640N, gp41 H643Y, gp41 M687I, gp41 S762N, Nef G12R, Nef A27V, Nef R35Q, Nef Q73X, Nef D186N; Isolate C substitutions: MA V35I, NC M46I, Pro M36I, Pro P79S, RT V179A, Vif N19D, Vpr Q11X, Vpr A59V, Vpr R62K, Vpr Q65X, Rev Q36R, Rev G93E, gp120 V38I, gp120 T278M, gp120 G410E, gp41 T626M, gp41 K655M, gp41 M687I, gp41 R729G, gp41 G786R, gp41 L851X, Nef R19K, Nef D186N. (B) An image of the ethidium bromide-stained agarose gel containing *vif-vpr* products (with *vif*: 2391 bp or without *vif*: 2161 bp) of the indicated A3G resistant isolates. Proviral DNAs were recovered from CEM-GFP cells infected with the resistant isolates produced in CEM2n cells and amplified with a primer set RSH1451/1454. Proviral plasmids (pIIIB Vif WT and Vif-null) and H_2O are controls. (C) Mutation matrices derived from sequence analysis of each A3G resistant viral isolate.
