# Supplementary material for: HIV-1 adaptation studies reveal a novel Env-mediated homeostasis mechanism for evading lethal hypermutation by APOBEC3G
Source: PLoS Pathog. 2018 Apr 20;14(4):e1007010. doi: 10.1371/journal.ppat.1007010 (PMC5931688; doi:10.1371/journal.ppat.1007010)
Supplement: S2 Fig — (A) Representative spreading infection assays in CEM-SS stably expressing a vector control or A3G (2 independent clones). (B) Immunoblots of A3G and TUB in the indicated cell lines. (C) Representative spreading infection assays in CEM-SS stably expressing a vector control or A3F (2 independent clones). (D) Immunoblots of A3F and TUB in the indicated cell lines. (PDF) [file ppat.1007010.s002.pdf]

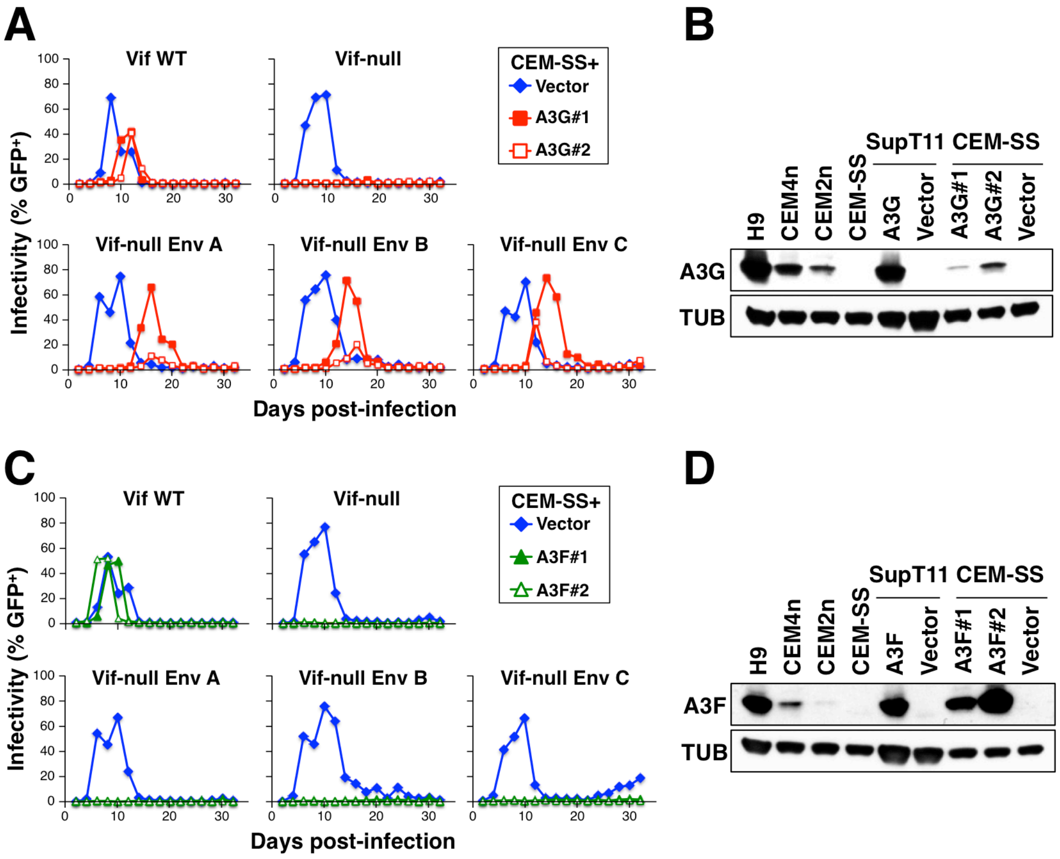

S2 Fig. Spreading infection phenotypes of Vif-null Env variant molecular clones. (A) Representative spreading infection assays in CEM-SS stably expressing a vector control or A3G (2 independent clones). (B) Immunoblots of A3G and TUB in the indicated cell lines. (C) Representative spreading infection assays in CEM-SS stably expressing a vector control or A3F (2 independent clones). (D) Immunoblots of A3F and TUB in the indicated cell lines.
