# Supplementary material for: HIV-1 adaptation studies reveal a novel Env-mediated homeostasis mechanism for evading lethal hypermutation by APOBEC3G
Source: PLoS Pathog. 2018 Apr 20;14(4):e1007010. doi: 10.1371/journal.ppat.1007010 (PMC5931688; doi:10.1371/journal.ppat.1007010)
Supplement: S3 Fig — Total luminescence signals of Vif-proficient, WT Env virus treated with DMSO, EFV or EFV plus C34. Each signal is normalized to Gag expression, and reported as relative to the DMSO treatment. The fusion-dependent signal (EFV) falls to background level (mean; <-1 x 10^−4^) by the additional C34 treatment. Each histogram bar represents the mean +/- SEM of 4 biologically independent experiments. (PDF) [file ppat.1007010.s003.pdf]

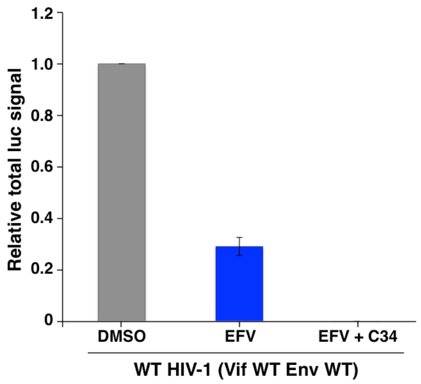

S3 Fig. EFV-resistant luciferase signal is completely eliminated by fusion inhibitor peptide C34. Total luminescence signals of Vif-proficient, WT Env virus treated with DMSO, EFV or EFV plus C34. Each signal is normalized to Gag expression, and reported as relative to the DMSO treatment. The fusion-dependent signal (EFV) falls to background level (mean; $<-1 \times 10^{-4}$) by the additional C34 treatment. Each histogram bar represents the mean +/- SEM of 4 biologically independent experiments.
