# Supplementary material for: HIV-1 adaptation studies reveal a novel Env-mediated homeostasis mechanism for evading lethal hypermutation by APOBEC3G
Source: PLoS Pathog. 2018 Apr 20;14(4):e1007010. doi: 10.1371/journal.ppat.1007010 (PMC5931688; doi:10.1371/journal.ppat.1007010)
Supplement: S4 Fig — (A) Cell-cell fusion assay schematic showing that GFP fluorescence only occurs upon reconstitution of the split GFP fragments in the same cytosol after fusion. Assay based on original studies [62, 66]. (B) Surface Env levels of HeLa cells transfected with the indicated viral constructs (flow cytometry data normalized to Vif-proficient condition for comparison). (C) Relative fusion efficiency of HeLa cells expressing the indicated HIV-1 constructs with TZM-bl target cells. Each experiment in panels B-C was done in triplicate and the average +/- SEM is shown for each condition. Statistical comparisons were done using a one-way ANOVA and Fisher’s LSD test (p-values above each panel). (PDF) [file ppat.1007010.s004.pdf]

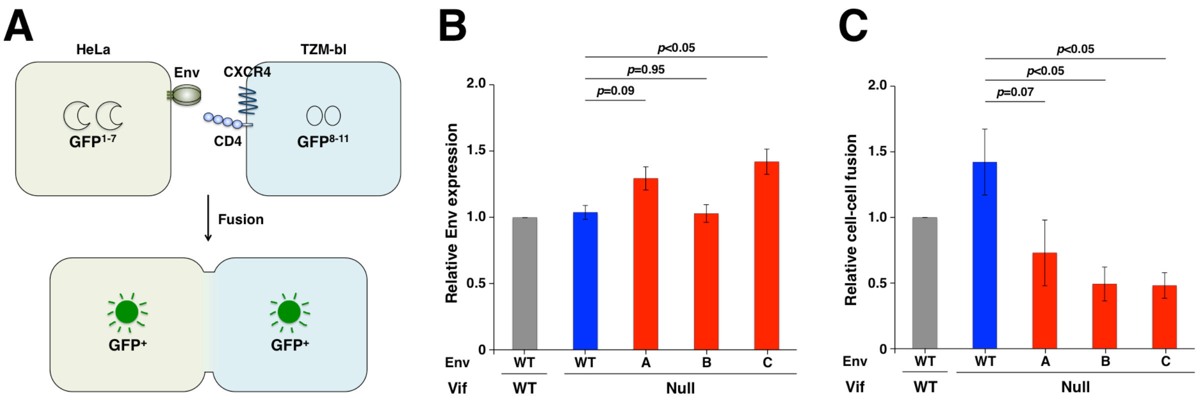

S4 Fig. Env variants exhibit reduced syncytium formation. (A) Cell-cell fusion assay schematic showing that GFP fluorescence only occurs upon reconstitution of the split GFP fragments in the same cytosol after fusion. Assay based on original studies in Kondo et al., 2010 and Symeonides et al., 2014. (B) Surface Env levels of HeLa cells transfected with the indicated viral constructs (flow cytometry data normalized to Vif-proficient condition for comparison). (C) Relative fusion efficiency of HeLa cells expressing the indicated HIV-1 constructs with TZM-bl target cells. Each experiment in panels B-C was done in triplicate and the average +/- SEM is shown for each condition. Statistical comparisons were done using a one-way ANOVA and Fisher's LSD test (*p*-values above each panel).
