# Supplementary material for: HIV-1 adaptation studies reveal a novel Env-mediated homeostasis mechanism for evading lethal hypermutation by APOBEC3G
Source: PLoS Pathog. 2018 Apr 20;14(4):e1007010. doi: 10.1371/journal.ppat.1007010 (PMC5931688; doi:10.1371/journal.ppat.1007010)
Supplement: S5 Fig — (A) Total luminescence normalized to Gag expression and reported relative to the Vif-proficient virus. Each histogram bar represents the mean +/- SEM of the normalized data from 3 biologically independent experiments (p-values above each panel from one-way ANOVA and Fisher’s LSD test). (B) Luminescence signal attributable to cell-cell fusion or virus transmission for the indicated viruses. Cell-cell fusion events are quantified as the fraction of total luciferase signal that is resistant to EFV-treatment, and virus transmission events are quantified by subtracting the cell-cell fusion signal from the total luminescence signal. Each histogram bar represents the mean +/- SEM of 3 biologically independent experiments (p-values above each panel from one-way ANOVA and Fisher’s LSD test). (PDF) [file ppat.1007010.s005.pdf]

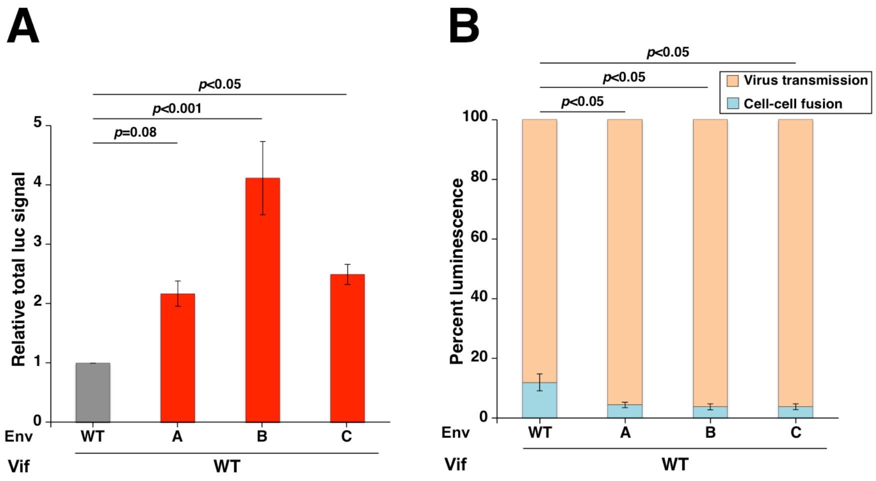

S5 Fig. Env mutations also reduce syncytium formation in the Vif-proficient context. (A) Total luminescence normalized to Gag expression and reported relative to the Vif-proficient virus. Each histogram bar represents the mean +/- SEM of the normalized data from 3 biologically independent experiments (*p*-values above each panel from one-way ANOVA and Fisher's LSD test). (B) Luminescence signal attributable to cell-cell fusion or virus transmission for the indicated viruses. Cell-cell fusion events are quantified as the fraction of total luciferase signal that is resistant to EFV-treatment, and virus transmission events are quantified by subtracting the cell-cell fusion signal from the total luminescence signal. Each histogram bar represents the mean +/- SEM of 3 biologically independent experiments (*p*-values above each panel from one-way ANOVA and Fisher's LSD test).
