# Supplementary material for: HIV-1 adaptation studies reveal a novel Env-mediated homeostasis mechanism for evading lethal hypermutation by APOBEC3G
Source: PLoS Pathog. 2018 Apr 20;14(4):e1007010. doi: 10.1371/journal.ppat.1007010 (PMC5931688; doi:10.1371/journal.ppat.1007010)
Supplement: S6 Fig — A representative single-cycle assay in 293T cells. The histogram reports mean +/- SD infectivity of 3 technically independent experiments. The immunoblots below show the indicated proteins in viral particles and cell extracts. (PDF) [file ppat.1007010.s006.pdf]

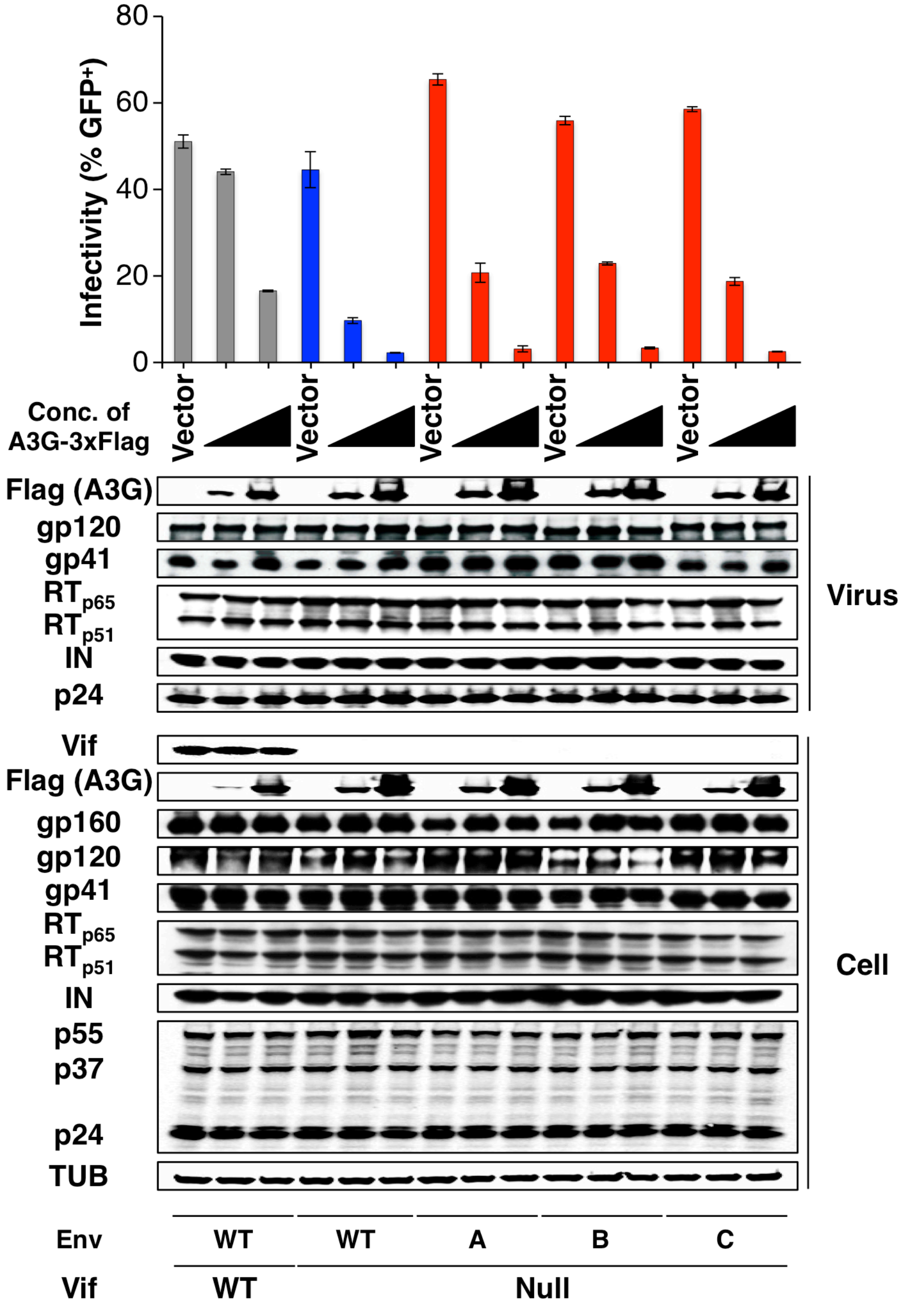

S6 Fig. Env adaptations do not confer resistance to A3G in adherent 293T cells. A representative single-cycle assay in 293T cells. The histogram reports mean +/- SD infectivity of 3 technically independent experiments. The immunoblots below show the indicated proteins in viral particles and cell extracts.
