# Supplementary material for: HIV-1 adaptation studies reveal a novel Env-mediated homeostasis mechanism for evading lethal hypermutation by APOBEC3G
Source: PLoS Pathog. 2018 Apr 20;14(4):e1007010. doi: 10.1371/journal.ppat.1007010 (PMC5931688; doi:10.1371/journal.ppat.1007010)
Supplement: S7 Fig — (A) Representative viral mutation plots from 1 of 3 independent experiments. The indicated viruses were produced in SupT11-A3G cells and used to infect CEM-GFP cells, from which the pol region was amplified by high-fidelity PCR, cloned, and sequenced. (B) Actual distribution of G-to-A mutations in the indicated dinucleotide contexts in 10 independent 564 bp pol region DNA sequences from panel A. (PDF) [file ppat.1007010.s007.pdf]

A

Vif WT

	A	C	G	T
**A**		0	**2**	0
**C**	0		0	3
**G**	2	0		0
**T**	1	0	0	

GA; 0%, GG; 100%
GT; 0%, GC; 0%
5640 bp sequenced
0.4 G→A mutations/kb

Vif-null

	A	C	G	T
**A**		0	**55**	0
**C**	0		0	2
**G**	5	0		0
**T**	0	0	0	

GA; 7%, GG; 91%
GT; 2%, GC; 0%
5640 bp sequenced
9.8 G→A mutations/kb

To

Vif-null Env A

	A	C	G	T
**A**		0	**15**	0
**C**	1		0	0
**G**	3	0		3
**T**	0	1	0	

GA; 33%, GG; 60%
GT; 0%, GC; 7%
5640 bp sequenced
2.7 G→A mutations/kb

Vif-null Env B

	A	C	G	T
**A**		0	**12**	0
**C**	0		0	2
**G**	3	0		0
**T**	0	0	0	

GA; 17%, GG; 83%
GT; 0%, GC; 0%
5640 bp sequenced
2.1 G→A mutations/kb

Vif-null Env C

	A	C	G	T
**A**		1	**12**	0
**C**	0		0	3
**G**	2	0		0
**T**	0	1	0	

GA; 8%, GG; 92%
GT; 0%, GC; 33%
5640 bp sequenced
2.1 G→A mutations/kb

From

B

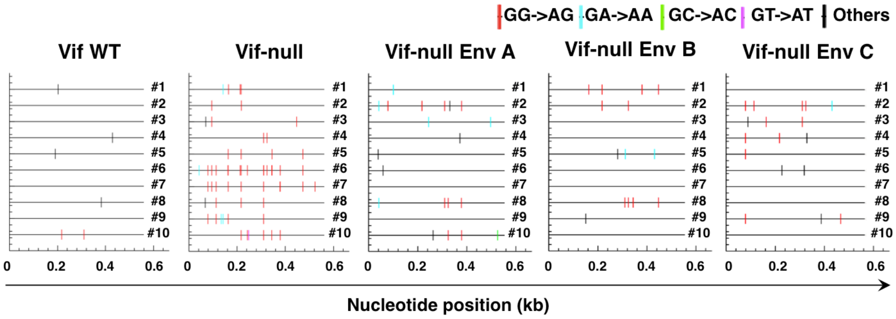

S7 Fig. Env adaptations protect Vif-null HIV-1 from A3G mutagenesis. (A) Representative viral mutation plots from 1 of 3 independent experiments. The indicated viruses were produced in SupT11-A3G cells and used to infect CEM-GFP cells, from which the *pol* region was amplified by high-fidelity PCR, cloned, and sequenced. (B) Actual distribution of G-to-A mutations in the indicated dinucleotide contexts in 10 independent 564 bp *pol* region DNA sequences from panel A.
