# Supplementary material for: HIV-1 adaptation studies reveal a novel Env-mediated homeostasis mechanism for evading lethal hypermutation by APOBEC3G
Source: PLoS Pathog. 2018 Apr 20;14(4):e1007010. doi: 10.1371/journal.ppat.1007010 (PMC5931688; doi:10.1371/journal.ppat.1007010)
Supplement: S8 Fig — (A) Representative viral mutation plots from 1 of 4 independent ERT experiments. The pol region was amplified by high-fidelity PCR, cloned, and sequenced. (B) Actual distribution of G-to-A mutations in the indicated dinucleotide contexts in 10 independent 564 bp pol region DNA sequences from panel A. (PDF) [file ppat.1007010.s008.pdf]

A

Vif WT

	A	C	G	T
A		1	**2**	1
C	0		0	2
G	1	0		0
T	0	0	0	

GA; 50%, GG; 50%
GT; 0%, GC; 0%
5640 bp sequenced
0.4 G→A mutations/kb

Vif-null

	A	C	G	T
A		0	**63**	0
C	0		0	2
G	1	0		0
T	1	0	0	

GA; 6%, GG; 94%
GT; 0%, GC; 0%
5640 bp sequenced
11.2 G→A mutations/kb

To

Vif-null Env A

	A	C	G	T
A		0	**53**	0
C	0		0	2
G	4	0		0
T	0	0	0	

GA; 17%, GG; 83%
GT; 0%, GC; 0%
5640 bp sequenced
9.4 G→A mutations/kb

Vif-null Env B

	A	C	G	T
A		1	**31**	0
C	0		0	0
G	1	0		0
T	0	1	0	

GA; 0%, GG; 100%
GT; 0%, GC; 0%
5640 bp sequenced
5.5 G→A mutations/kb

Vif-null Env C

	A	C	G	T
A		0	**38**	0
C	0		0	0
G	4	0		0
T	0	2	0	

GA; 16%, GG; 84%
GT; 0%, GC; 0%
5640 bp sequenced
6.7 G→A mutations/kb

From

B

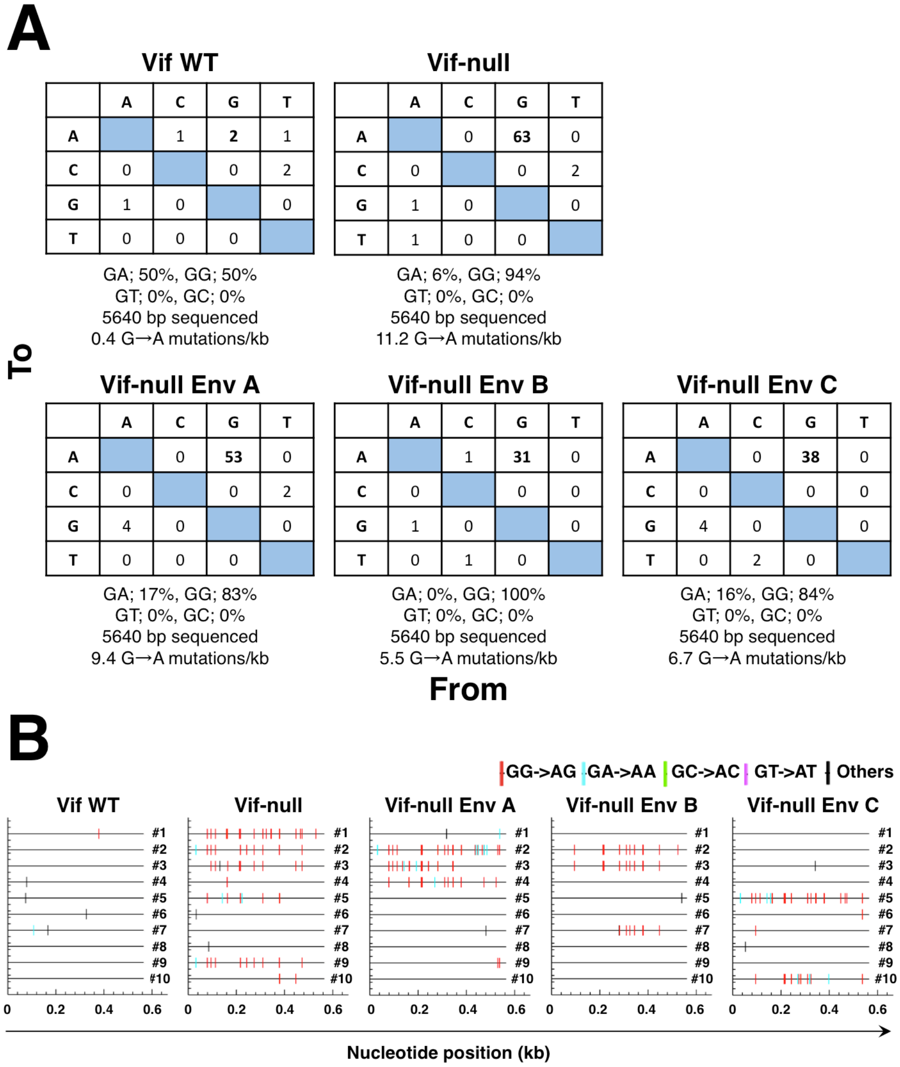

S8 Fig. Endogenous reverse transcription data. (A) Representative viral mutation plots from 1 of 4 independent ERT experiments. The *pol* region was amplified by high-fidelity PCR, cloned, and sequenced. (B) Actual distribution of G-to-A mutations in the indicated dinucleotide contexts in 10 independent 564 bp *pol* region DNA sequences from panel A.
