# Supplementary material for: HIV-1 adaptation studies reveal a novel Env-mediated homeostasis mechanism for evading lethal hypermutation by APOBEC3G
Source: PLoS Pathog. 2018 Apr 20;14(4):e1007010. doi: 10.1371/journal.ppat.1007010 (PMC5931688; doi:10.1371/journal.ppat.1007010)
Supplement: S9 Fig — (A) Immunoblots of the indicated proteins in viral particles and SupT11-vector producer cells from one representative experiment. (B) Relative RT packaging into viral particles produced in SupT11-vector cells. RT packaging levels were quantified based on band intensity and normalized by each p24 of the virions. Each data point is the average +/- SD of biological triplicates. (C) Relative RT activity in viral particles produced in SupT11-vector cells. RT activity in each viral lysate normalized for p24 contents was measured. Each data point is the average +/- SD of biological triplicates. (D-G) Kinetics of early RT, late RT, 2-LTR circle, and proviral DNA during infection of CEM-GFP cells using viruses originally produced in SupT11-vector cells (mean +/- SD of 3 biologically independent experiments). Statistical comparisons were done using Student’s t test (p-values above each panel in comparison to Vif-null HIV-1; *: p<0.05, **: p<0.01, ***: p<0.001). (PDF) [file ppat.1007010.s009.pdf]

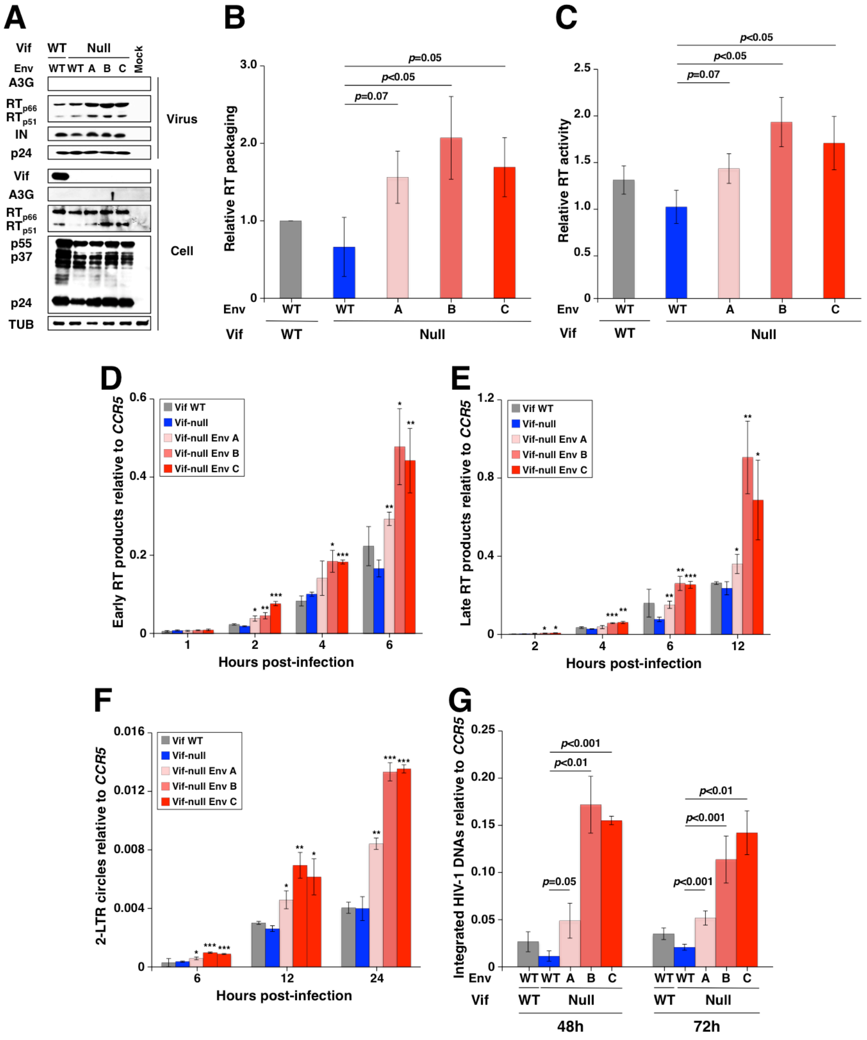

S9 Fig. RT packaging and RT kinetics of Env variants produced in SupT11-vector cells. (A) Immunoblots of the indicated proteins in viral particles and SupT11-vector producer cells from one representative experiment. (B) Relative RT packaging into viral particles produced in SupT11-vector cells. RT packaging levels were quantified based on band intensity and normalized by each p24 of the virions. Each data point is the average +/- SD of biological triplicates. (C) Relative RT activity in viral particles produced in SupT11-vector cells. RT activity in each viral lysate normalized for p24 contents was measured. Each data point is the average +/- SD of biological triplicates. (D-G) Kinetics of early RT, late RT, 2-LTR circle, and proviral DNA during infection of CEM-GFP cells using viruses originally produced in SupT11-vector cells (mean +/- SD of 3 biologically independent experiments). Statistical comparisons were done using Student's t test (*p*-values above each panel in comparison to Vif-null HIV-1; *: $p<0.05$, **: $p<0.01$, ***: $p<0.001$).
