# Supplementary material for: HIV-1 adaptation studies reveal a novel Env-mediated homeostasis mechanism for evading lethal hypermutation by APOBEC3G
Source: PLoS Pathog. 2018 Apr 20;14(4):e1007010. doi: 10.1371/journal.ppat.1007010 (PMC5931688; doi:10.1371/journal.ppat.1007010)
Supplement: S1 Table — (PDF) [file ppat.1007010.s010.pdf]

S1 Table. Summary of SupT11 spreading infection data.

HIV-1 IIIB molecular clone	Day of peak (% GFP^+ at peak)		
	Vector	A3G	A3F
Series 1[1]			
Vif WT	10±2 (48±9)	12±2 (47±14)	14±3 (49±14)
Vif-null	12±2 (71±10)[2]	NS[5]	NS[5]
Vif-null Env A	7±1 (74±4)[3]	15±1 (45±19)[3]	NS[5]
Vif-null Env B	7±1 (77±6)[3]	12±1 (61±14)[4]	NS[5]
Vif-null Env C	7±1 (66±9)[3]	14±1 (63±12)[2]	NS[5]
Series 2[6]			
Vif WT	8 (27)	12 (28)	12 (35)
Vif-null	12 (54)	NS[5]	NS[5]
Vif-null gp120 A58V	8 (28)	12 (20)	NS[2]
Vif-null gp120 P79L/S143N	8 (67)	12 (24)	NS[5]
Vif-null gp120 M426L/Q442P	12 (29)	NS[5]	NS[5]
Vif-null gp41 T626M	8 (61)	20 (11)	NS[5]
Vif-null gp41 H643Y	12 (34)	20 (20)	NS[5]
Vif-null gp41 K655M	8 (72)	16 (46)	NS[5]
Vif-null gp41 M687I	12 (55)	24 (17)[7]	NS[5]
Vif-null gp41 V822I	8 (65)	28 (13)[7]	NS[5]

[1]Virus infectivity quantified every 2 days for >4 weeks (mean +/- SD of 14 biologically independent experiments).

[2]$p<0.01$, [3]$p<0.001$, [4]$p>0.05$. Student's t-test comparisons with Vif WT day of peak data.

[5]No significant virus replication (<5% GFP^+).

[6]Virus infectivity from one representative experiment quantified every 4 days for >4 weeks.

[7]Variable day of peak and % GFP^+.
