# Supplementary material for: HIV-1 adaptation studies reveal a novel Env-mediated homeostasis mechanism for evading lethal hypermutation by APOBEC3G
Source: PLoS Pathog. 2018 Apr 20;14(4):e1007010. doi: 10.1371/journal.ppat.1007010 (PMC5931688; doi:10.1371/journal.ppat.1007010)
Supplement: S2 Table — (PDF) [file ppat.1007010.s011.pdf]

S2 Table. Summary of CEM-SS spreading infection data.

HIV-1 IIIB molecular clone	Day of peak (% GFP^{+})[1]		
Series 1			
	Vector	A3G#1	A3G#2
Vif WT	8±0 (51±11)	12±1 (47±7)	12±0 (48±12)
Vif-null	9±1 (66±9)[2]	NS[5]	NS[5]
Vif-null Env A	9±1 (68±4)[2]	16±0 (62±2)[3]	17±1 (9±2)[3]
Vif-null Env B	9±1 (72±5)[2]	14±1 (66±13)[4]	16±1 (16±4)[3]
Vif-null Env C	9±1 (67±2)[2]	13±1 (72±4)[2]	12±0 (29±5)[2]
Series 2			
	Vector	A3F#1	A3F#2
Vif WT	8±0 (45±15)	9±1 (46±4)	8±0 (41±14)
Vif-null	9±1 (73±3)[2]	NS[5]	NS[5]
Vif-null Env A	9±1 (67±7)[2]	NS[5]	NS[5]
Vif-null Env B	9±1 (73±2)[2]	NS[5]	NS[5]
Vif-null Env C	9±1 (64±4)[2]	NS[5]	NS[5]

[1]Virus infectivity quantified every 2 days for >4 weeks (mean +/- SD of 4 biologically independent experiments).

[2]$p>0.05$, [3]$p<0.001$, [4]$p<0.05$. Student's t-test comparisons with Vif WT day of peak data.

[5]No significant virus replication (<5% GFP^{+}).
