# Supplementary material for: HIV-1 adaptation studies reveal a novel Env-mediated homeostasis mechanism for evading lethal hypermutation by APOBEC3G
Source: PLoS Pathog. 2018 Apr 20;14(4):e1007010. doi: 10.1371/journal.ppat.1007010 (PMC5931688; doi:10.1371/journal.ppat.1007010)
Supplement: S3 Table — (PDF) [file ppat.1007010.s012.pdf]

S3 Table. Oligonucleotide sequences	
Name	Sequences (5' to 3')
RSH1431	GAA GCT TGC TCG GCT CTT AG
RSH1432	AAG ACA AGG ACC AAA GGA ACC
RSH1433	GGA TTT TCA GGC CCA ATT TT
RSH1434	TGG ATG GCC CAA AAG TTA AA
RSH1435	CAT GCT TCC CAT GTT TCC TT
RSH1440	GAT ACT TGG GCA GGA GTG GA
RSH1441	TAT GGG AAT TGG CTC AAA GG
RSH1442	GCA TAA GAG GTA AGG TGC AGA AA
RSH1443	CCT CAA TAG CCC TCA GCA AA
RSH1444	GAC GGT ACA GGC CAG ACA AT
RSH1445	GGG AGT GAA TTA GCC CTT CC
RSH1451	CAA GGC CAA TGG ACA TAT CA
RSH1452	TTT GCT GGT CCT TTC CAA AC
RSH1454	CAA ACT TGG CAA TGA AAG CA
RSH1649	GGA CAG ATA GGG TTA TAG AA
RSH2456	GGT CTC TCT GGT TAG ACC AG
RSH4068	GAA TTA GTT GGT CAT TTT ACT AAT CTT TTC CAT GTG TTA ATC CTC
RSH4069	GGA AAA GAT TAG TAA AAT GAC CAA CTA ATT CAT CTG CAC TAT TTT
RSH4196	TCC ART ATT TRC CAT AAA RAA AAA
RSH4197	TTY AGA TTT TTA AAT GGY TYT TGA
RSH4205	AAT ATT CCA RTR TAR CAT RAC AAA AAT
RSH4206	AAT GGY TYT TGA TAA ATT TGA TAT GT
RSH7418	CTG GTC TAA CCA GAG AGA CC
RSH7808	GTC GAC ATA GCA GAA TAG GCG TT
RSH7809	CAT ATG CTT TAG CAT CTG ATG CA
RSH7813	CAT ATG ATA CAG AGG TAC ATA ATG TTT GGG
RSH7814	GCT AGCT ATC TGT TTT AAA GTG GCA
RSH7815	CCC ACA GAC CTC AAC CCA CAA

RSH7816	TTG TGG GTT GAG GTC TGT GGG
RSH7817	GCT AGC AAA TTA AGA GAA CAA TTT GG
RSH7818	GGA TCC GTT CAC TAA TCG AAT GG
RSH7819	AAA ATT ATT CAT AAT AAT AGT AGG AGG CTT G
RSH7820	CAA GCC TCC TAC TAT TAT TAT GAA TAA TTT T
RSH8373	NNN NNG ATA TCA CCA TGA AGC CTC ACT TCA GAA ACA CAG TG
RSH8374	NGC GGC CGC CCG TTT TCC TGA TTC TGG AGA ATG GCC CG
RSH8086	GGA TCC TTA GCA CTT ATC TGG GA
RSH8087	CTC GAG ATG CTG CTC CCA C
RSH8088	CAG CTA TAG CAA TAG CTG AGG GG
RSH8089	CCC CTC AGC TAT TGC TAT AGC TG
RSH9972	GCC TCA ATA AAG CTT GCC TTG A
RSH9973	TGA CTA AAA GGG TCT GAG GGA TCT
RSH9974	FAM-AGA GTC ACA CAA CAG ACG GGC ACA CAC TA-TAMRA
RSH9975	TGT GTG CCC GTC TGT TGT GT
RSH9976	GAG TCC TGC GTC GAG AGA GC
RSH9977	FAM-CAG TGG CGC CCG AAC AGG GA-TAMRA
RSH9978	AAC TAG GGA ACC CAC TGC TTA AG
RSH9979	TCC ACA GAT CAA GGA TAT CTT GTC
RSH9980	FAM-ACA CTA CTT GAA GCA CTC AAG GCA AGC TTT-TAMRA
RSH9981	TGC TGG GAT TAC AGG CGT GAG
RSH9982	CCA GAA GAG CTG AGA CAT CCG
RSH9983	GCC AAG CAG CTG AGA GGT TAC T
RSH9984	FAM-TCC CCT ACA AGA AAC TCT CCC CGG-TAMRA
RSH10734	FAM-AAA TAT CCC AAA GAG AGA-TAMRA
